# Supplementary material for: The tetraspanin CD9 controls migration and proliferation of parietal epithelial cells and glomerular disease progression
Source: Nat Commun. 2019 Jul 24;10:3303. doi: 10.1038/s41467-019-11013-2 (PMC6656772; doi:10.1038/s41467-019-11013-2)
Supplement: Supplementary file 1 — Supplementary Information [file 41467_2019_11013_MOESM1_ESM.pdf]

Supplementary Information

The tetraspanin CD9 controls invasive migration and proliferation of parietal epithelial cells and glomerular disease progression

Lazareth et al.

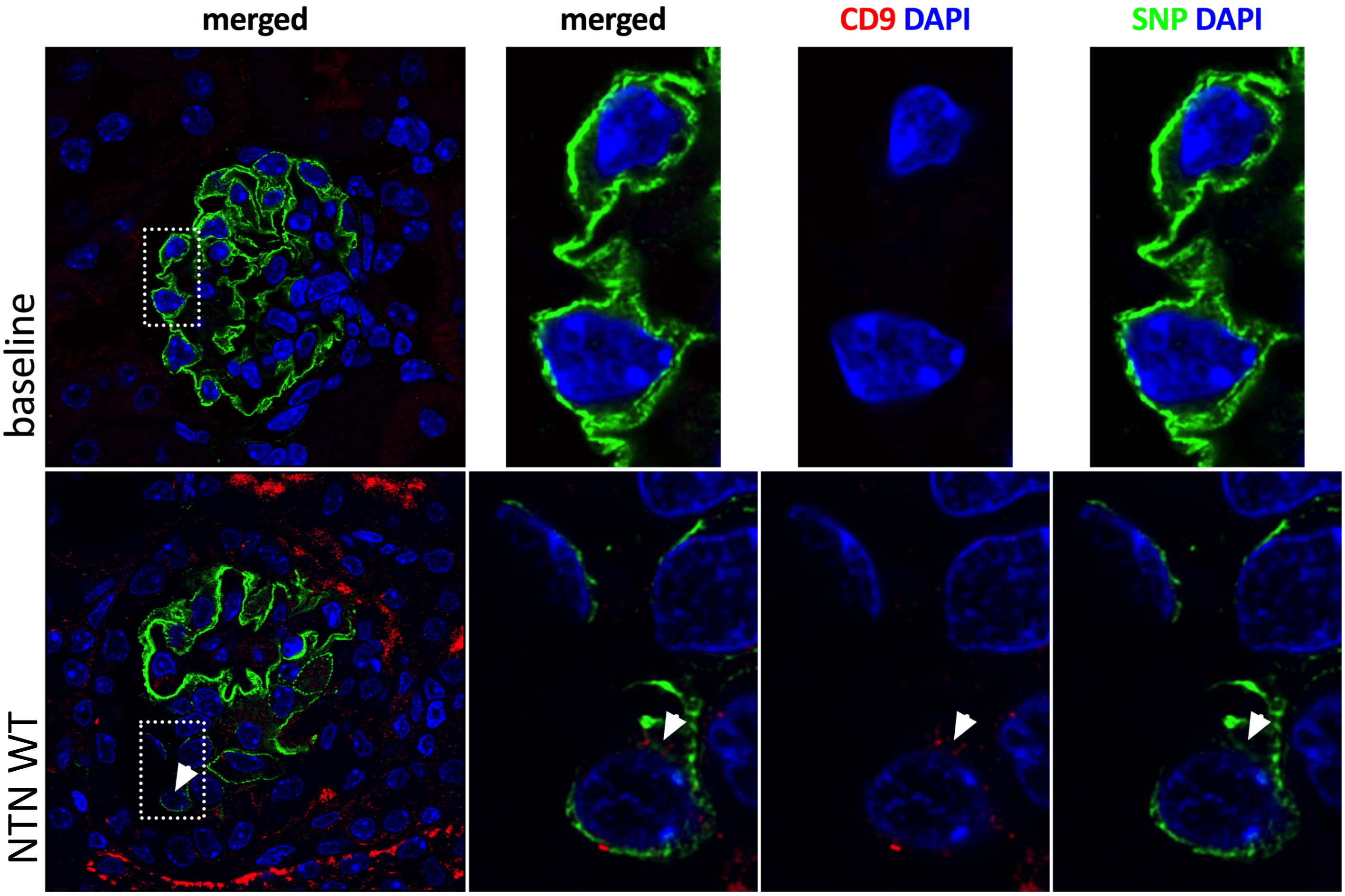

<u>Supplementary figure 1</u>: ***De novo* CD9 expression is weak in podocytes in NTN in mice.** Representative images of immunofluorescent stainings for CD9 (red) and SNP (green) in WT and WT NTN mice. Nuclei were counterstained with DAPI (blue). CD9 expression in podocytes is weak at baseline and in the NTN model.

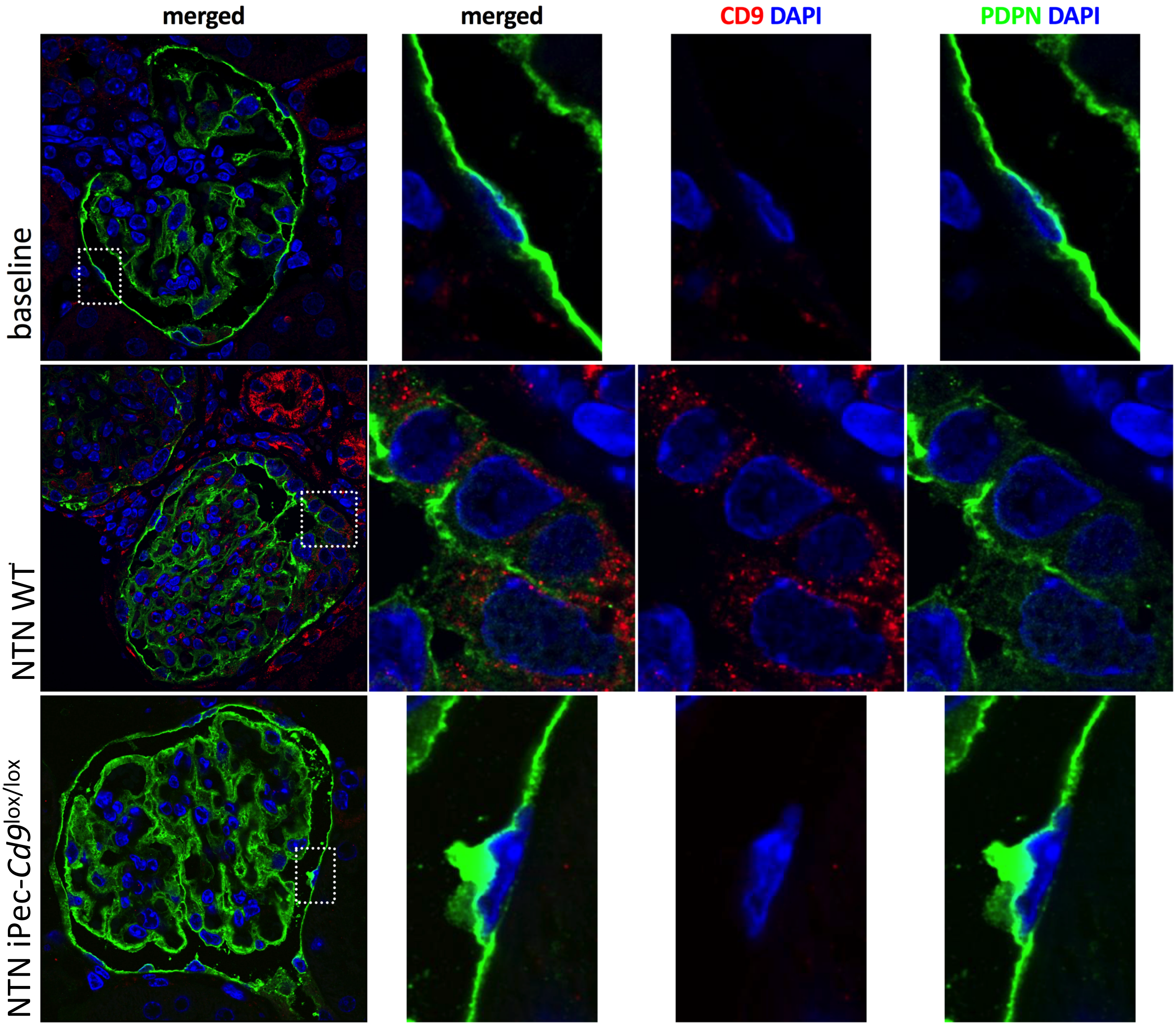

<u>Supplementary figure 2</u>: NTN induces *De novo* CD9 expression in PECs in mice. Representative images of immunofluorescent stainings for CD9 (red) and PDPN (green) in WT, WT NTN and iPec-*Cd9*$^{lox/lox}$ NTN mice. Nuclei were counterstained with DAPI (blue).

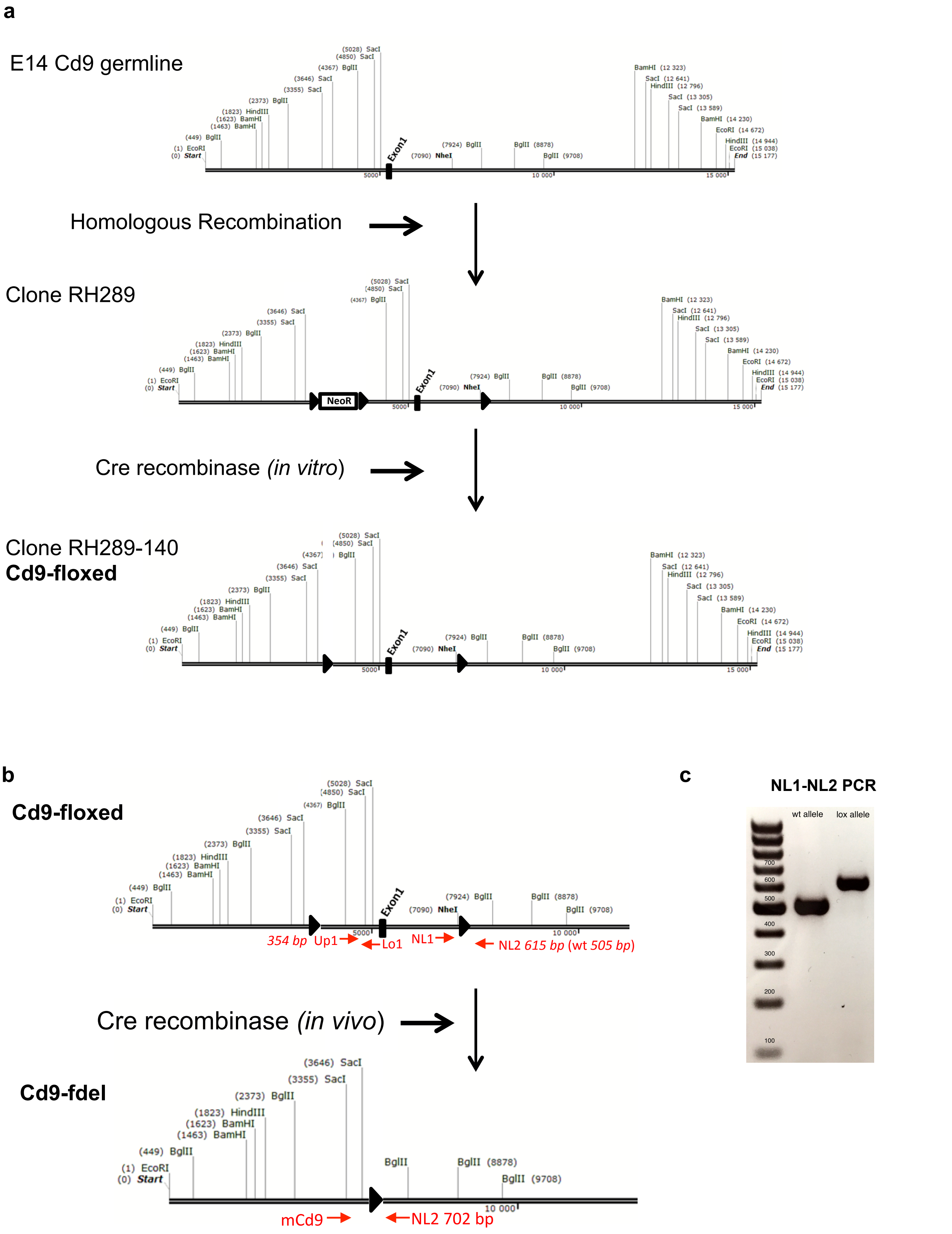
a
E14 Cd9 germline
(0) Start
(1) EcoRI
(449) BglII
(1463) BamHI
(1623) BamHI
(1823) HindIII
(2373) BglII
(3355) SacI
(3646) SacI
(4367) BglII
(4850) SacI
(5028) SacI
Exon1
(7090) NheI
(7924) BglII
BglII (8878)
BglII (9708)
BamHI (12 323)
SacI (12 641)
HindIII (12 796)
SacI (13 305)
SacI (13 589)
BamHI (14 230)
EcoRI (14 672)
HindIII (14 944)
EcoRI (15 038)
End (15 177)
5000
10 000
15 000
Homologous Recombination
Clone RH289
NeoR
Cre recombinase (in vitro)
Clone RH289-140
Cd9-floxed
b
Cd9-floxed
354 bp Up1
Lo1
NL1
NL2 615 bp (wt 505 bp)
Cre recombinase (in vivo)
Cd9-fdel
BglII
mCd9
NL2 702 bp
c
NL1-NL2 PCR
wt allele
lox allele
700
600
500
400
300
200
100

<u>Supplementary figure 3</u> : ***Cd9*^floxed^ mice construction.**

(a) *In vitro* conditional homologous recombination of the mouse *Cd9* gene - Strategy

Germline configuration of the *Cd9* gene encompassing Exon 1 between 2 EcoRI restriction sites (upper panel). On the PTV-0 vector, a 9kb genomic fragment containing exon 1 (starting at bp -1625 from the ATG site) and a lox site introduced at the first NheI site of intron 1 (3.6 kb 3' from the start of the 9 kb fragment) was inserted 3' to the Neo^r gene and a short arm of 787 bp was inserted 5' to the Neo^r gene (middle panel). The recombination was performed in E14 ES cells. One of the selected ES clones, RH289, was transfected with the expression vector pIC-Cre to remove the Neo^r gene (lower panel).

(b) *In vivo* conditional homologous recombination of the mouse *Cd9* gene - Genotyping

Upper panel shows mapping of Cd9 floxed gene before *in vivo* Cre recombination. The different primers used for genotyping (Up1 and Lo1 for 5' sequence genotyping, NL1 and NL2 for exon1 floxed genotyping) are also shown. Lower panel shows mapping of Cd9 deleted gene (Cd9-fdel) after action of Cre recombination. Primers for genotyping are also shown on graph (mCd9 and NL2). (c) Photomicrographs of agarose gel migration showing PCR products for *Cd9* floxed (615 bp) and *wt* genotype (505 bp) before Cre recombination (NL1-NL2 PCR).

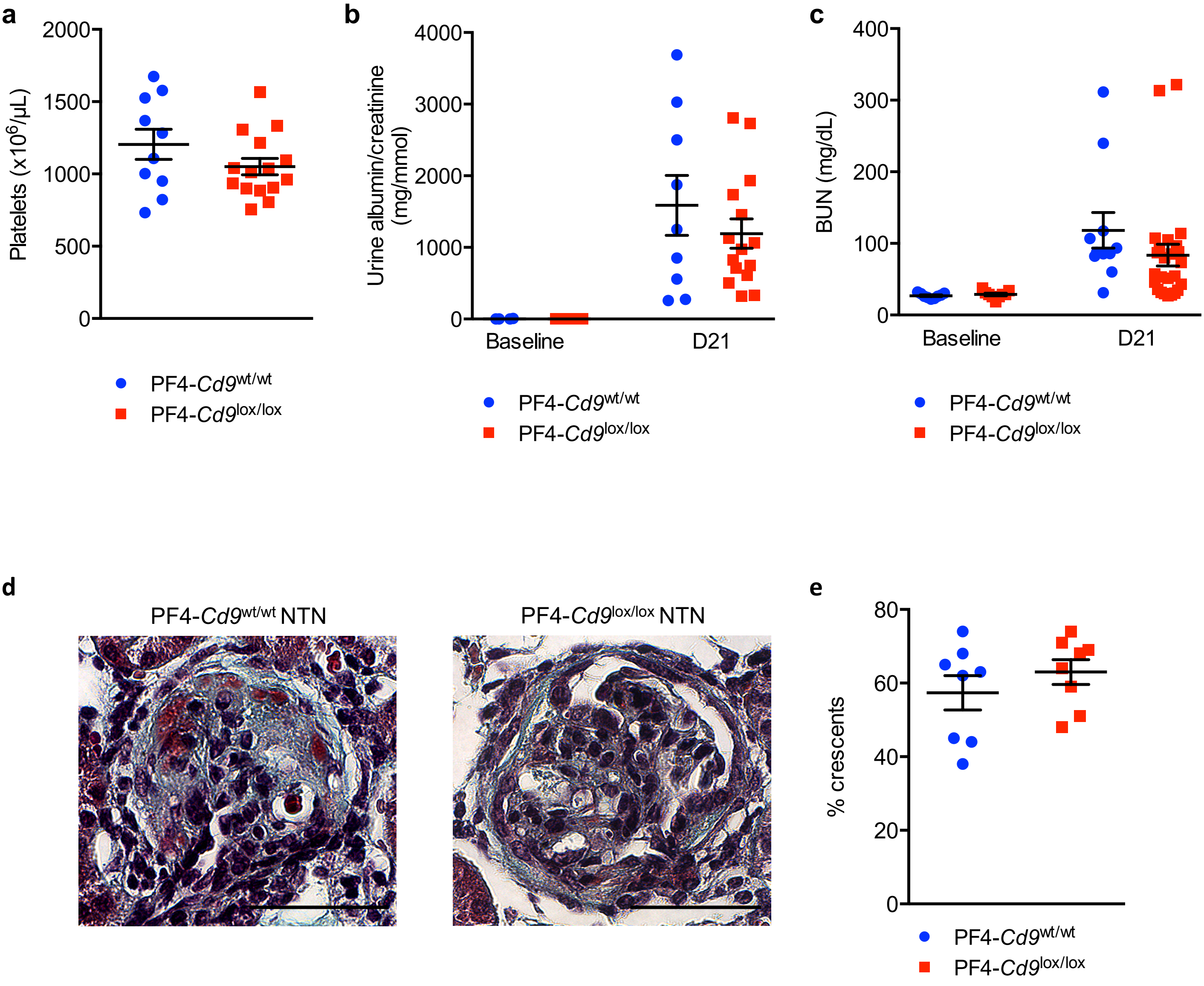

<u>Supplementary figure 4</u> : Platelet-selective CD9 depletion does not protects mice from NTN-induced RPGN.

(a) Platelets count in PF4-*Cd9*$^{wt/wt}$ and PF4-*Cd9*$^{lox/lox}$ mice at baseline (n=10 PF4-*Cd9*$^{wt/wt}$ and n=15 PF4-*Cd9*$^{lox/lox}$ mice). (b-c) Urine albumin-to-creatinine ratio (b) and Blood Urea Nitrogen (BUN) (c) in PF4-*Cd9*$^{wt/wt}$ and PF4-*Cd9*$^{lox/lox}$ mice at baseline and after 21 days of NTN model (n=5 mice at baseline, n=9 PF4-*Cd9*$^{wt/wt}$ at D21 and n=15 PF4-*Cd9*$^{lox/lox}$ at D21). (d) Representative images showing Masson's trichrome staining on kidney sections from PF4-*Cd9*$^{wt/wt}$ and PF4-*Cd9*$^{lox/lox}$ mice after 21 days of NTN model. Scale bar, 50µm. (g) Associated quantification of the percentage of crescents (n=8 mice per group). (a-c, e) Data represent mean +/- s.e.m. and individual values are shown in dots. Source data are provided as a Source Data file.

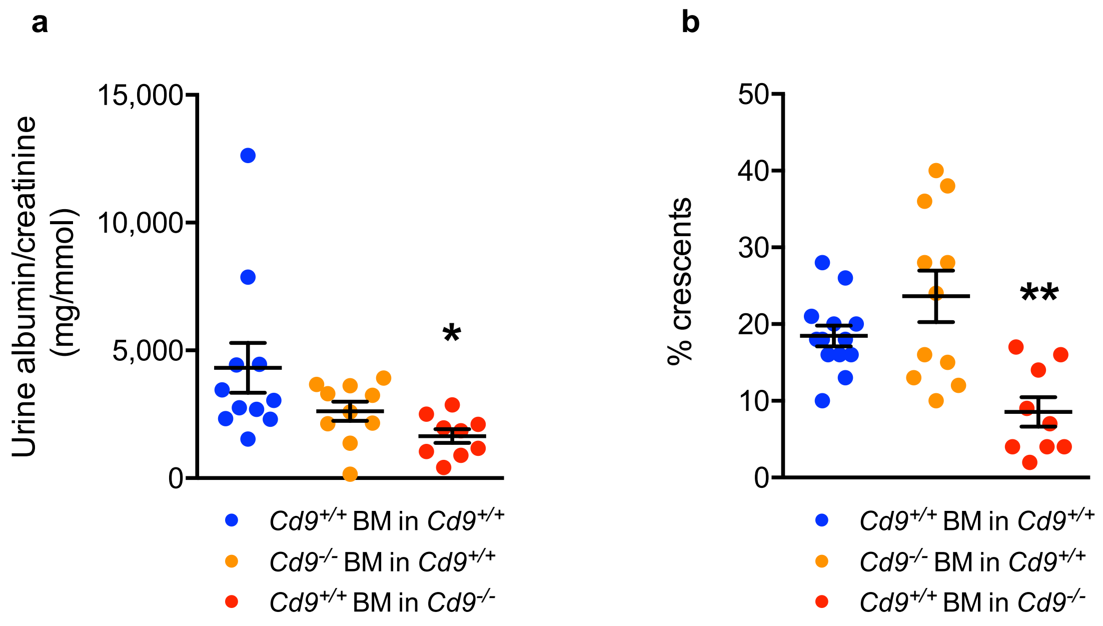

<u>**Supplementary figure 5**</u> : **CD9 deficiency in kidneys but in bone marrow protects mice from NTN-induced CGN.**

(a) Urine albumin-to-creatinine ratio after 10 days of NTN model in *Cd9*$^{+/+}$ and *Cd9*$^{-/-}$ mice after bone marrow (BM) transplantation. * P<0.05 between *Cd9*$^{+/+}$ BM in *Cd9*$^{+/+}$ mice and *Cd9*$^{+/+}$ BM in *Cd9*$^{-/-}$ mice, *t* test. (*Cd9*$^{+/+}$ BM in *Cd9*$^{+/+}$ mice: n=11, *Cd9*$^{-/-}$ BM in *Cd9*$^{+/+}$ mice: n=9, and *Cd9*$^{+/+}$ BM in *Cd9*$^{-/-}$mice: n=9). (b) Percentage of crescents (*Cd9*$^{+/+}$ BM in *Cd9*$^{+/+}$ mice: n=11, *Cd9*$^{-/-}$ BM in *Cd9*$^{+/+}$ mice: n=9, and *Cd9*$^{+/+}$ BM in *Cd9*$^{-/-}$mice: n=9). **P<0.01 between *Cd9*$^{+/+}$ BM in *Cd9*$^{+/+}$ mice and *Cd9*$^{+/+}$ BM in *Cd9*$^{-/-}$ mice, *t* test. Data represent mean +/- s.e.m. and individual values are shown in dots. Source data are provided as a Source Data file.

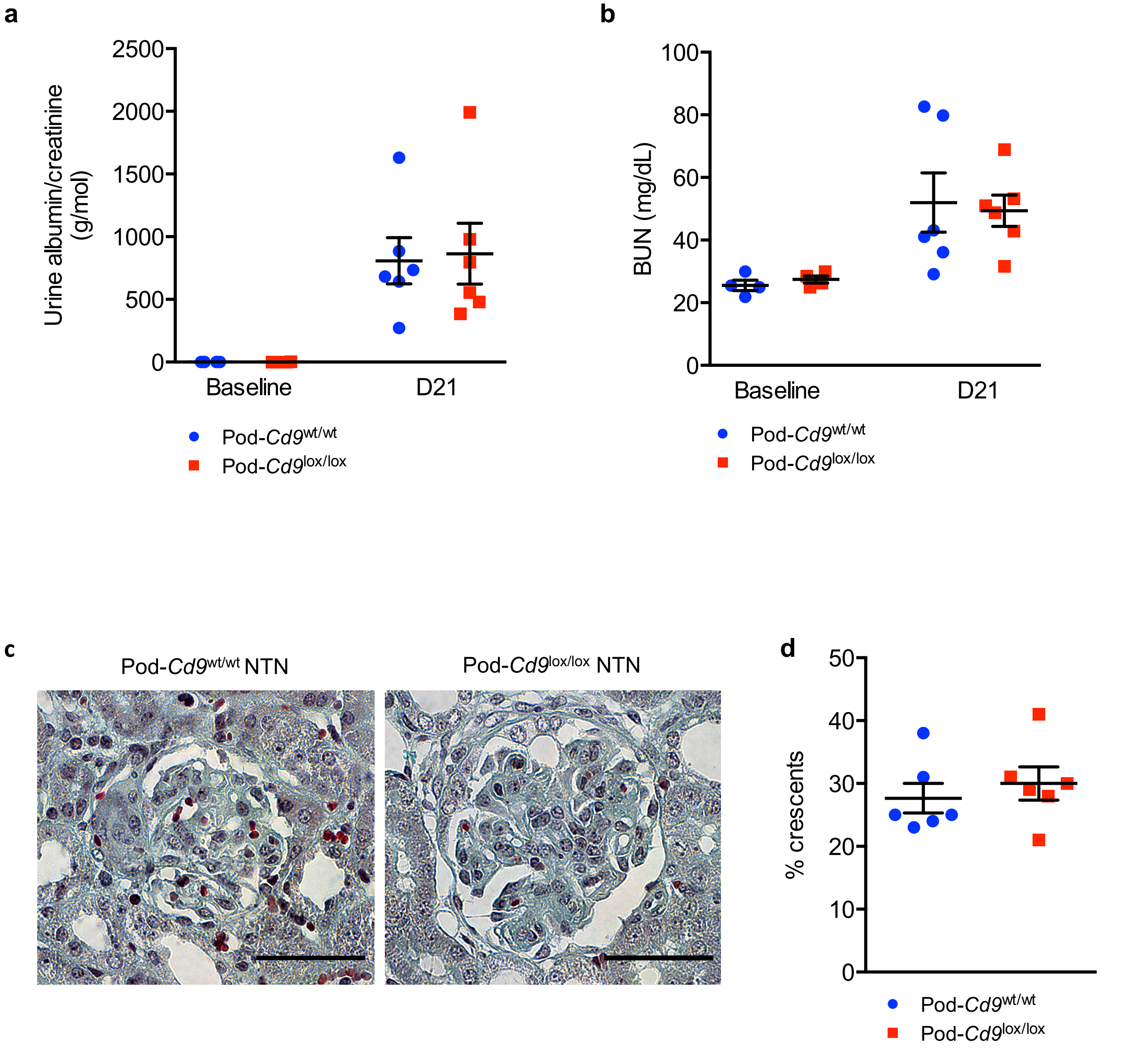

Supplementary figure 6: **Podocyte-selective CD9 depletion does not protects mice from NTN-induced RPGN.**

(a-b) Urine albumin-to-creatinine ratio (a) and Blood Urea Nitrogen (BUN) (b) in Pod-*Cd9*$^{wt/wt}$ and Pod-*Cd9*$^{lox/lox}$ mice at baseline (n=4 mice per group) and after 21 days of NTN model (n=6 mice per group). (c) Representative images showing Masson's trichrome staining on kidney sections from Pod-*Cd9*$^{wt/wt}$ and Pod-*Cd9*$^{lox/lox}$ mice after 21 days of NTN model. Scale bar, 50µm. (d) Associated quantification of the percentage of crescents (n=6 mice per group). (a, b, d) Data represent mean +/- s.e.m. and individual values are shown in dots. Source data are provided as a Source Data file.

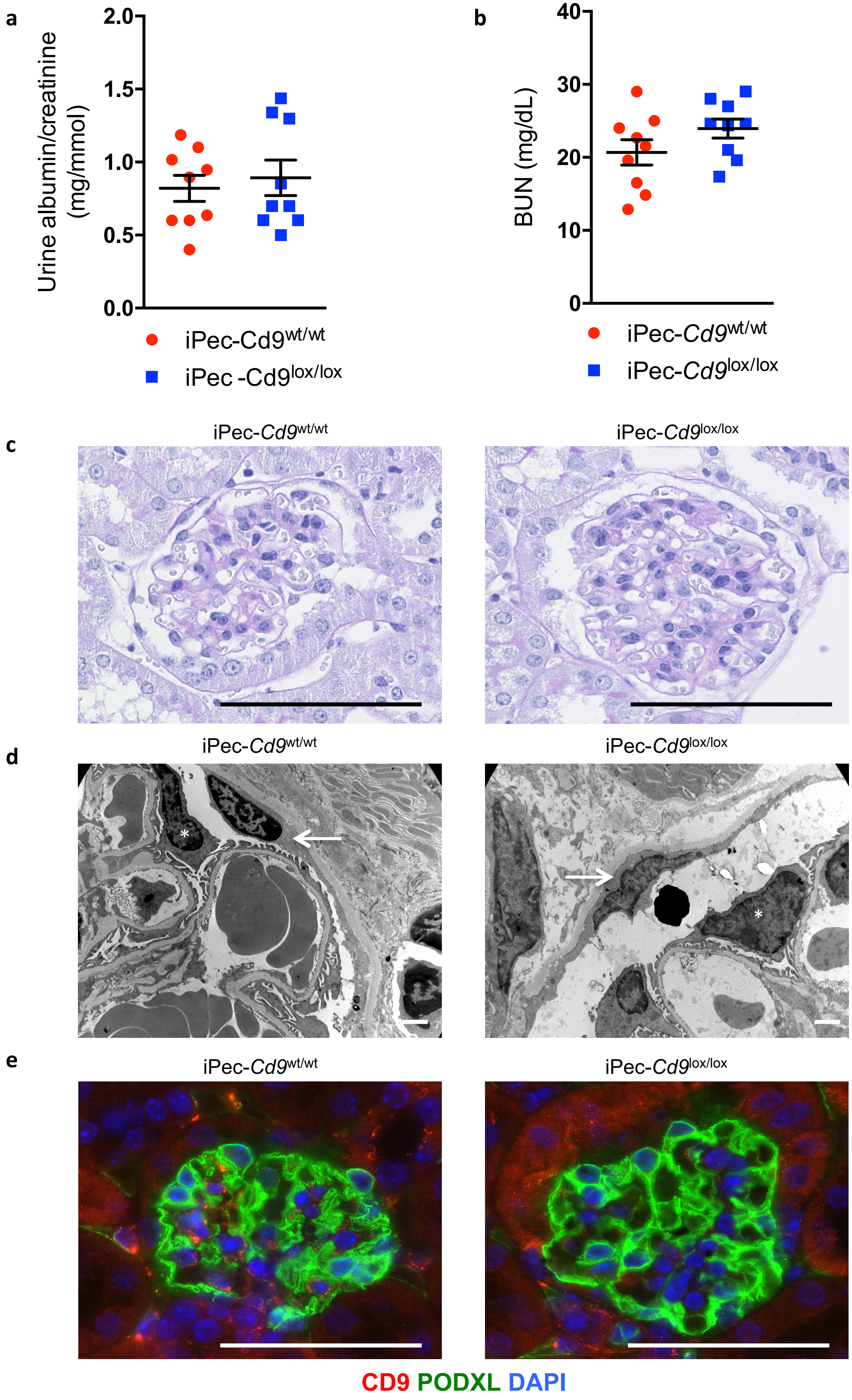
a
Urine albumin/creatinine (mg/mmol)
2.0
1.5
1.0
0.5
0.0
iPec-Cd9wt/wt
iPec -Cd9lox/lox
b
BUN (mg/dL)
40
30
20
10
0
iPec-Cd9wt/wt
iPec-Cd9lox/lox
c
iPec-Cd9wt/wt
iPec-Cd9lox/lox
d
iPec-Cd9wt/wt
iPec-Cd9lox/lox
e
iPec-Cd9wt/wt
iPec-Cd9lox/lox
CD9 PODXL DAPI

Supplementary figure 7: PEC-selective CD9 depletion does not influence renal function nor PEC phenotype at baseline.

(a-b) Urine albumin-to-creatinine ratio (a) and Blood Urea Nitrogen (BUN) (b) in iPec-*Cd9*$^{wt/wt}$ and iPec-*Cd9*$^{lox/lox}$ young adults (n=9 mice per group). (c) Representative images showing Periodic Acid-Shiff staining on kidney sections from iPec-*Cd9*$^{wt/wt}$ and iPec-*Cd9*$^{lox/lox}$ young adults. Scale bar, 50µm. (d) Representative images of transmission electron microscopy of glomerular ultrastructure in iPec-*Cd9*$^{wt/wt}$ and iPec-*Cd9*$^{lox/lox}$ mice. Arrows indicate parietal epithelial cells and * stands for podocytes. Scale bar, 1µm. (e) Representative images of immunofluorescent stainings for CD9 (red) and PODXL/podocalyxin (green) in iPec-*Cd9*$^{wt/wt}$ and iPec-*Cd9*$^{lox/lox}$ mice. Nuclei were stained with DAPI (blue). Scale bar, 50µm. Data represent mean +/- s.e.m. and individual values are shown in dots. Source data are provided as a Source Data file.

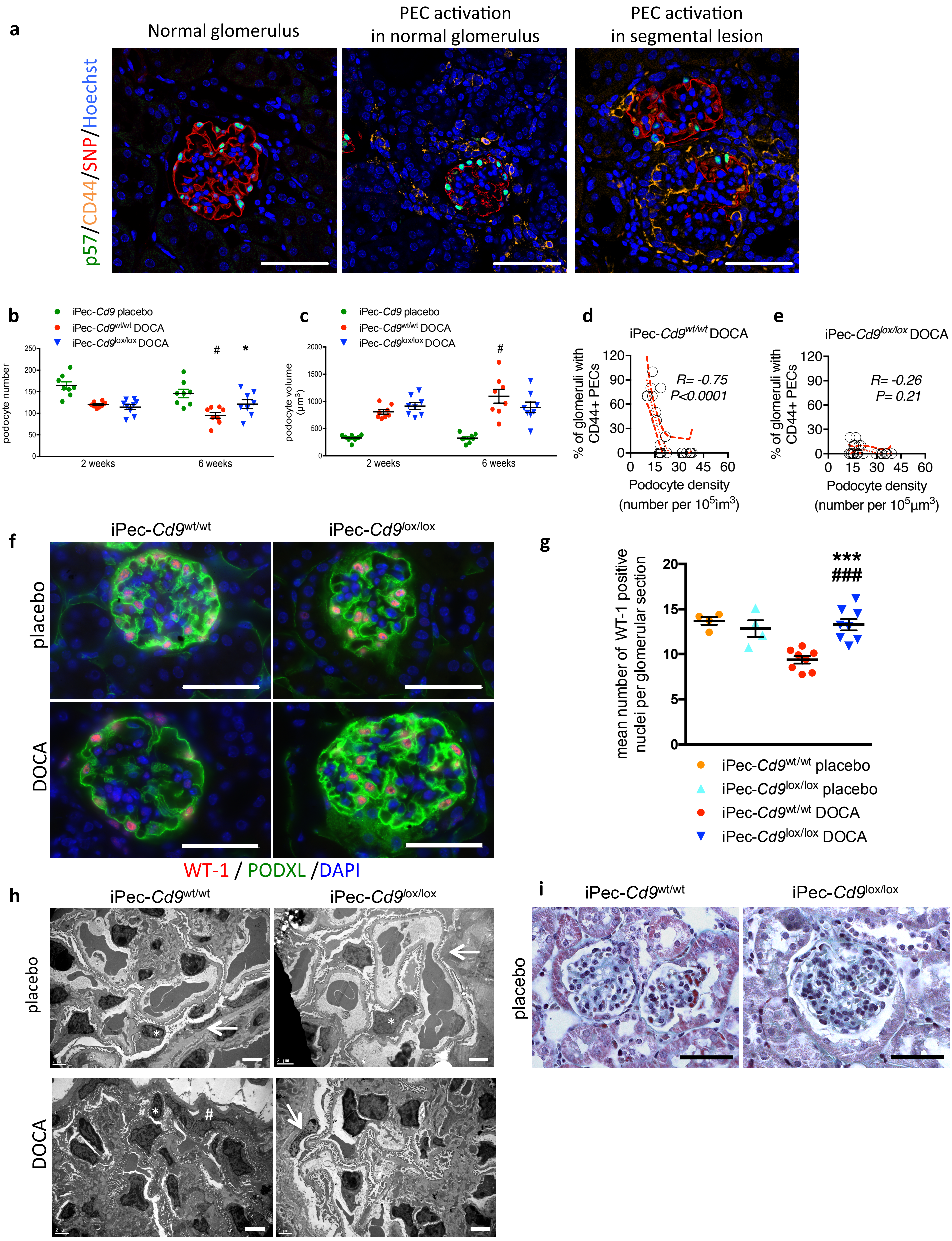

a
Normal glomerulus
PEC activation in normal glomerulus
PEC activation in segmental lesion
p57/CD44/SNP/Hoechst
b
iPec-Cd9 placebo
iPec-Cd9wt/wt DOCA
iPec-Cd9lox/lox DOCA
podocyte number
2 weeks
6 weeks
c
podocyte volume (µm3)
d
iPec-Cd9wt/wt DOCA
% of glomeruli with CD44+ PECs
R= -0.75
P<0.0001
Podocyte density (number per 10⁵ìm³)
e
iPec-Cd9lox/lox DOCA
R= -0.26
P= 0.21
Podocyte density (number per 10⁵µm³)
f
iPec-Cd9wt/wt
iPec-Cd9lox/lox
placebo
DOCA
WT-1 / PODXL /DAPI
g
mean number of WT-1 positive nuclei per glomerular section
***
###
iPec-Cd9wt/wt placebo
iPec-Cd9lox/lox placebo
iPec-Cd9wt/wt DOCA
iPec-Cd9lox/lox DOCA
h
iPec-Cd9wt/wt
iPec-Cd9lox/lox
placebo
DOCA
i
iPec-Cd9wt/wt
iPec-Cd9lox/lox
placebo

<u>Supplementary figure 8</u>: PEC-selective CD9 depletion protects mice from podocyte loss in the DOCA-salt model.

(a) Representative image of of immunofluorescent stainings for p57 (green), CD44 (orange) and synaptopodin (SNP, red) on normal kidney section, in case of PEC activation in a normal glomerulus and in segmental lesion. Nuclei were stained with Hoechst (blue). Scale bar, 50 µm. (b-c) Quantification of podocyte number (b) and podocyte volume (c) after 2 and 6 weeks of DOCA or placebo treatment in iPec-*Cd9*$^{wt/wt}$ and iPec-*Cd9*$^{lox/lox}$ mice. *t* test: * P<0.05 between iPec-*Cd9*$^{wt/wt}$ DOCA and iPec-*Cd9*$^{lox/lox}$ DOCA mice after 6 weeks; # P<0.05 between week 2 and week 6 in iPec-*Cd9*$^{wt/wt}$ mice. (d-e) Correlation between percentage of CD44 positive PEC and podocyte density in iPec-*Cd9*$^{wt/wt}$ (d)and iPec-*Cd9*$^{lox/lox}$ mice (e) after 6 weeks of DOCA or placebo treatment. (f) Representative images of immunofluorescent stainings for WT-1 (red) and PODXL/podocalyxin (green) on kidney sections from iPec-*Cd9*$^{wt/wt}$ and iPec-*Cd9*$^{lox/lox}$ mice after 6 weeks of DOCA or placebo treatment. Scale bar, 50µm. (g) Associated quantification of the mean number of WT-1 positive nuclei per glomerular area in iPec-*Cd9*$^{wt/wt}$ and iPec-*Cd9*$^{lox/lox}$. *t* test: *** P<0.05 between iPec-*Cd9*$^{wt/wt}$ DOCA and iPec-*Cd9*$^{lox/lox}$ DOCA mice. (n=4 mice in placebo groups, n=8 mice in DOCA groups). (h) Representative images of transmission electron microscopy of glomerular ultrastructure in iPec-*Cd9*$^{wt/wt}$ and iPec-*Cd9*$^{lox/lox}$ mice after 6 weeks of DOCA or placebo treatment. Scale bar, 2µm. Arrows indicate parietal epithelial cells; * stand for podocytes and # shows fibrous synechia. (i) Masson trichrome staining on kidney section in iPec-*Cd9* mice after 6 weeks of placebo treatment. Scale bar, 50 µm. Data represent mean +/- s.e.m. and individual values are shown in dots. Source data are provided as a Source Data file.

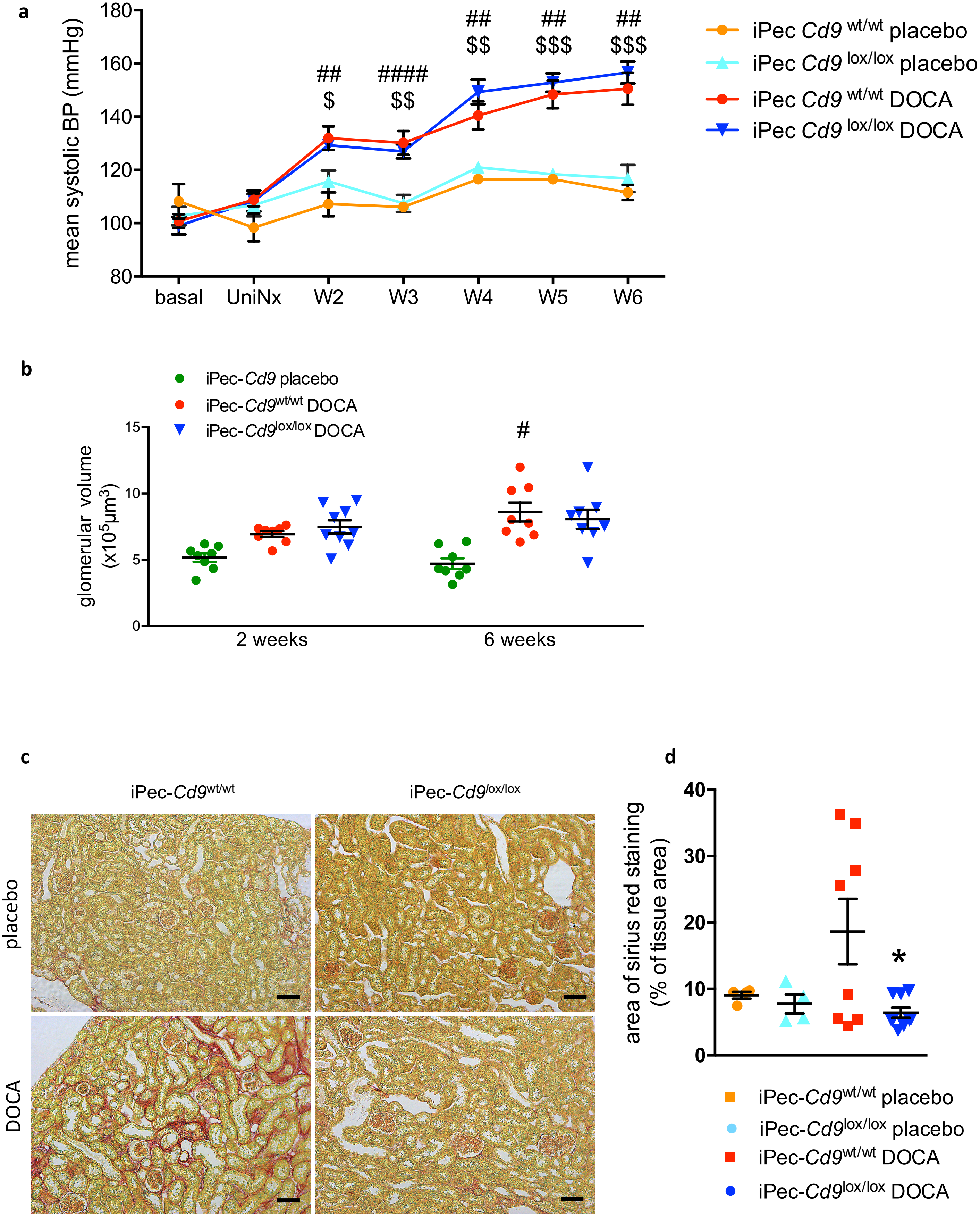
a
mean systolic BP (mmHg)
180
160
140
120
100
80
basal
UniNx
W2
W3
W4
W5
W6
##
$
####
$$
##
$$
##
$$$
##
$$$
iPec Cd9 wt/wt placebo
iPec Cd9 lox/lox placebo
iPec Cd9 wt/wt DOCA
iPec Cd9 lox/lox DOCA
b
iPec-Cd9 placebo
iPec-Cd9wt/wt DOCA
iPec-Cd9lox/lox DOCA
glomerular volume (x10^5µm^3)
15
10
5
0
#
2 weeks
6 weeks
c
iPec-Cd9wt/wt
iPec-Cd9lox/lox
placebo
DOCA
d
area of sirius red staining (% of tissue area)
40
30
20
10
0
*
iPec-Cd9wt/wt placebo
iPec-Cd9lox/lox placebo
iPec-Cd9wt/wt DOCA
iPec-Cd9lox/lox DOCA

<u>Supplementary figure 9</u>: **PEC-selective CD9 depletion protects mice from FSGS and fibrosis in an hypertension-independent manner in the DOCA-salt model.**

(a) Mean systolic blood pressure during DOCA salt model. ANOVA Statistical analyses between iPec-*Cd9*$^{wt/wt}$ placebo and DOCA mice: ## P<0.01; ####P<0.0001 and between iPec-*Cd9*$^{lox/lox}$ placebo and DOCA mice: $: P<0.05; $$: P<0.01; $$$: P<0.001 (UniNx = uninephrectomy). (n=4 mice in placebo groups, n=9 iPec-*Cd9*$^{wt/wt}$ DOCA and n=7 iPec-*Cd9*$^{lox/lox}$ DOCA mice) (b) Glomerular volume in iPec-*Cd9*$^{wt/wt}$ and iPec-*Cd9*$^{lox/lox}$ mice after 2 and 6 weeks of DOCA or placebo treatment. #P<0.05 between 2 and 6 weeks of DOCA treatment in iPec-*Cd9*$^{wt/wt}$ mice (n=8 mice per group). (c) Representative images of red sirius staining of kidney sections in iPec-*Cd9*$^{wt/wt}$ and iPec-*Cd9*$^{lox/lox}$ mice after 6 weeks of DOCA or placebo treatment. Scale bar, 50µm. (d) Associated quantification of the percentage of red sirius staining per tissue area. *t* test: * P<0.05 between iPec-*Cd9*$^{wt/wt}$ DOCA and iPec-*Cd9*$^{lox/lox}$ DOCA mice (n=4 mice in placebo groups, n=8 mice in DOCA groups). Data represent mean +/- s.e.m. and individual values are shown in dots. Source data are provided as a Source Data file.

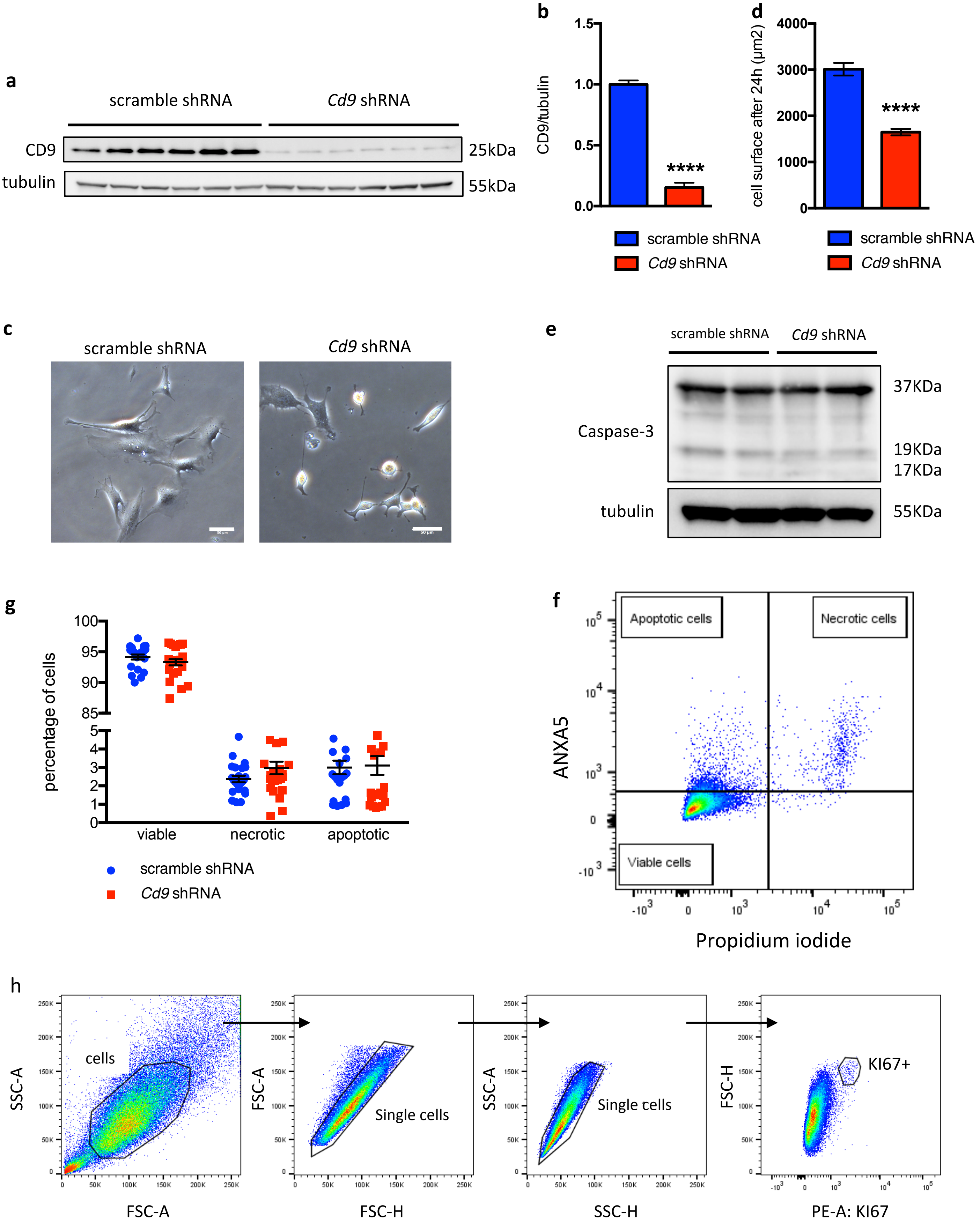
a
scramble shRNA
Cd9 shRNA
CD9
tubulin
25kDa
55kDa
b
CD9/tubulin
1.5
1.0
0.5
0.0
****
scramble shRNA
Cd9 shRNA
d
cell surface after 24h (µm2)
4000
3000
2000
1000
0
****
scramble shRNA
Cd9 shRNA
c
scramble shRNA
Cd9 shRNA
e
scramble shRNA
Cd9 shRNA
Caspase-3
tubulin
37KDa
19KDa
17KDa
55KDa
g
percentage of cells
100
95
90
85
5
4
3
2
1
0
viable
necrotic
apoptotic
scramble shRNA
Cd9 shRNA
f
Apoptotic cells
Necrotic cells
Viable cells
ANXA5
Propidium iodide
h
cells
Single cells
Single cells
KI67+
SSC-A
FSC-A
SSC-A
FSC-H
FSC-A
FSC-H
SSC-H
PE-A: KI67

<u>Supplementary figure 10</u>: *In vitro* CD9 deficiency in PEC modifies cell shape but does not modulate cell apoptosis

(a-b) Western Blot analysis (a) and associated quantification (b) of the expression of CD9 in PEC line after transduction with either scramble shRNA-coding lentiviral particles or *Cd9* shRNA-coding lentiviral particles. Tubulin is used as a loading control. **** P<0,0001. Data represent mean +/- s.e.m. of n=6 experiments. (c-d) Cell morphology (c) and cell surface (d) of control (scramble shRNA) and CD9-deficient (*Cd9* shRNA) PECs after 24 hours of adhesion. Scale bar, 50µm. *t* test: **** P<0.0001. Date represent mean +/- s.e.m. (n=237 cells for scramble shRNA and n=228 cells for *Cd9* shRNA). (e) Western Blot for total caspase-3 expression and tubulin in PEC line transduced with either scramble shRNA of *Cd9* shRNA at baseline. (f) Representative flow cytometry results for control (scramble shRNA) and CD9-deficient (*Cd9* shRNA) PEC after 24 hours of adhesion. The horizontal axis shows the fluorescence intensity of propidium iodide and the vertical axis shows that of ANXA5/annexin V-PE. (g) Quantification of the percentage of viable (Q1: ANXA5-negative IP-negative), apoptotic (Q1: ANXA5-positive IP-negative) and necrotic (Q2: ANXA5-positive IP-positive) cells. Values are means ± s.e.m of 30 wells/ condition in 2 independent experiments. (h) Flow cytometry gating strategy for KI67+ PECs. Single cells were gated on FSC-A/FSC-H then on SSC-A/SSC-H and KI67+ were selected on the fluorescence intensity for PE-A. Source data are provided as a Source Data file.

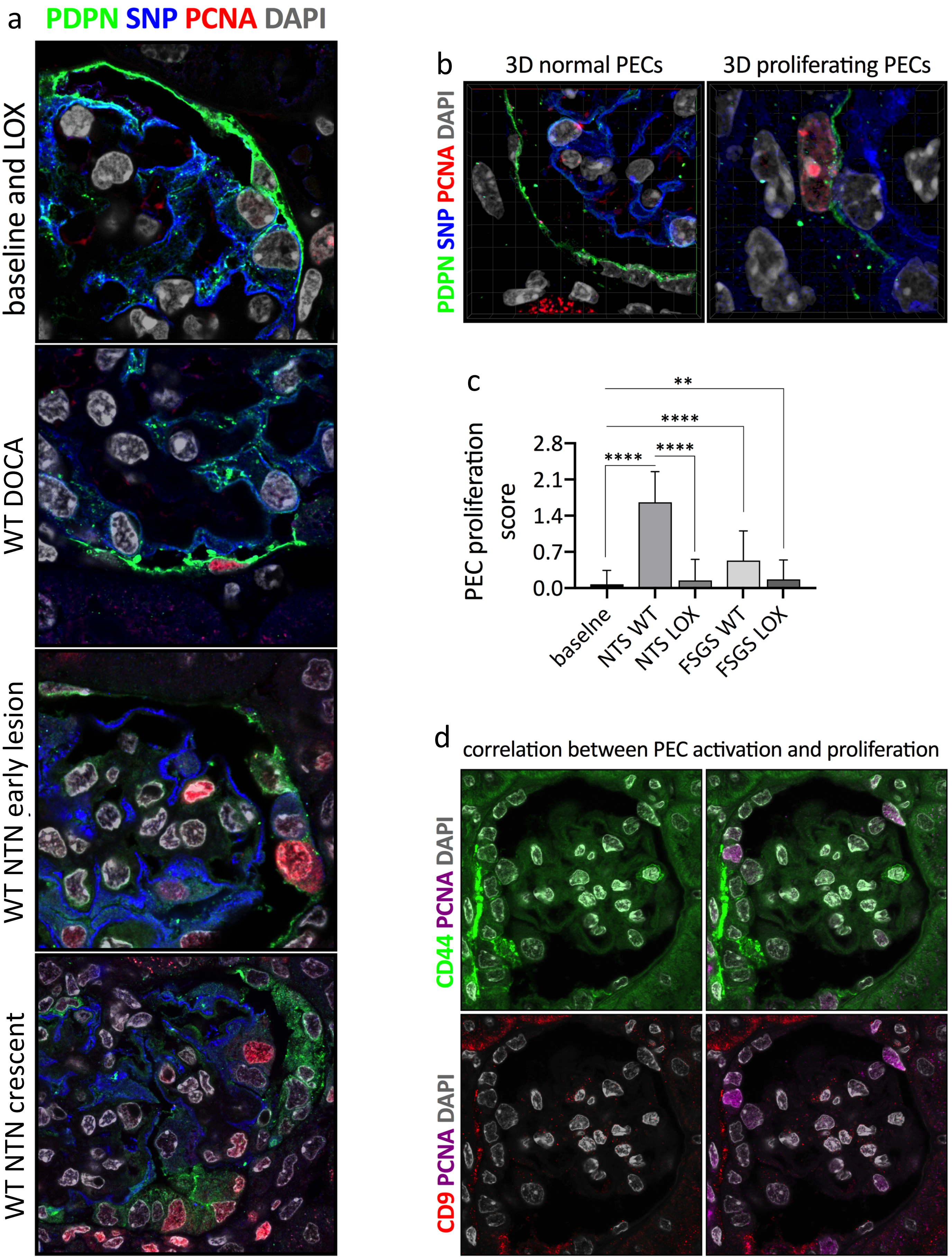
a
PDPN SNP PCNA DAPI
baseline and LOX
WT DOCA
WT NTN early lesion
WT NTN crescent
b
3D normal PECs
3D proliferating PECs
PDPN SNP PCNA DAPI
c
PEC proliferation score
2.8
2.1
1.4
0.7
0.0
**
****
****
****
baseline
NTS WT
NTS LOX
FSGS WT
FSGS LOX
d
correlation between PEC activation and proliferation
CD44 PCNA DAPI
CD9 PCNA DAPI

Supplementary figure 11: PEC proliferation is inhibited in iPec-*Cd9*$^{lox/lox}$ mice. (a) Representative images are provided showing normal non-proliferating PECs at baseline and in iPec-*Cd9*$^{lox/lox}$ mice, scattered PEC proliferation in a mouse model of FSGS, and in early crescentic lesions, and extensive PEC proliferation in cellular crescents. (b) 3D reconstructions provide strong evidence of co-localisation between a proliferation marker (PCNA), a PEC marker (podoplanin; PDPN) in the absence of a podocyte marker (synaptopodin; SNP). (c) Quantification of PEC proliferation using a score system per glomerulus, based on the classification of glomerular cross-sections based on the presence of zero PCNA+PDPN+ cells (score: 0), the presence of only one PCNA+PDPN+ cell (score: 1) or at least 2 PCNA+PDPN+ cells (score: 2) – this proliferation score clearly shows that NTS and FSGS lead to PEC proliferation, with a higher proliferative rate in NTS compared to FSGS, and that PEC-selective CD9 KO inhibits this proliferative response in both models. ANOVA: ** $p<0.01$, **** $p<0.0001$. Data represent mean +/- s.e.m. (d) Markers of PEC activation (CD44 and CD9) co-localize with PCNA in PECs during NTN. Source data are provided as a Source Data file.

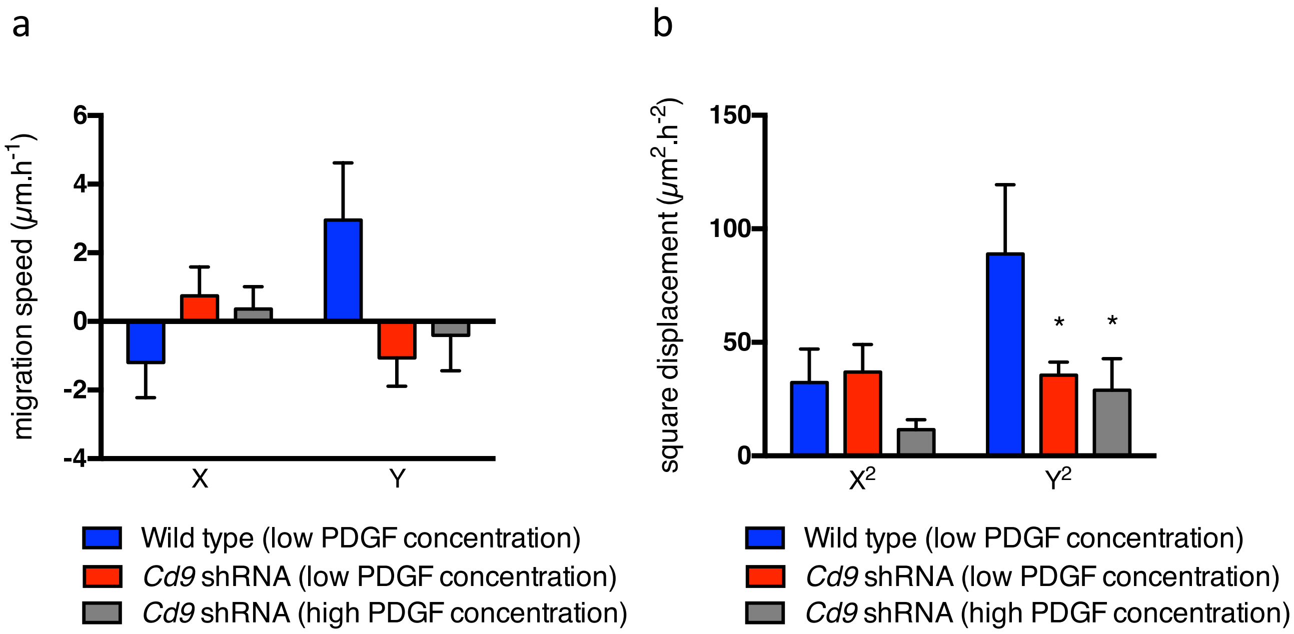

Supplementary figure 12: Average speed (a) and quadratic speed (a) of wild type and CD9 knocked-down PECs submitted to a PDGF gradient in a microchannel, either at low average PDGF concentration (10 ng.ml^{-1}, respectively in red (n=30) and blue (n=52)) or high average PDGF concentration (20 ng.ml^{-1}, grey (n=27)). X is the channel axis direction and Y the orthogonal axis, along which the gradient is produced. Data represent mean +/- s.e.m. and individual values are shown in dots. 2 way ANOVA with Sidak's multiple comparison test: * $p<0.05$ wild type vs. *Cd9* shRNA low PDGF and wild type vs. *Cd9* shRNA high PDGF on Y^2. Source data are provided as a Source Data file.

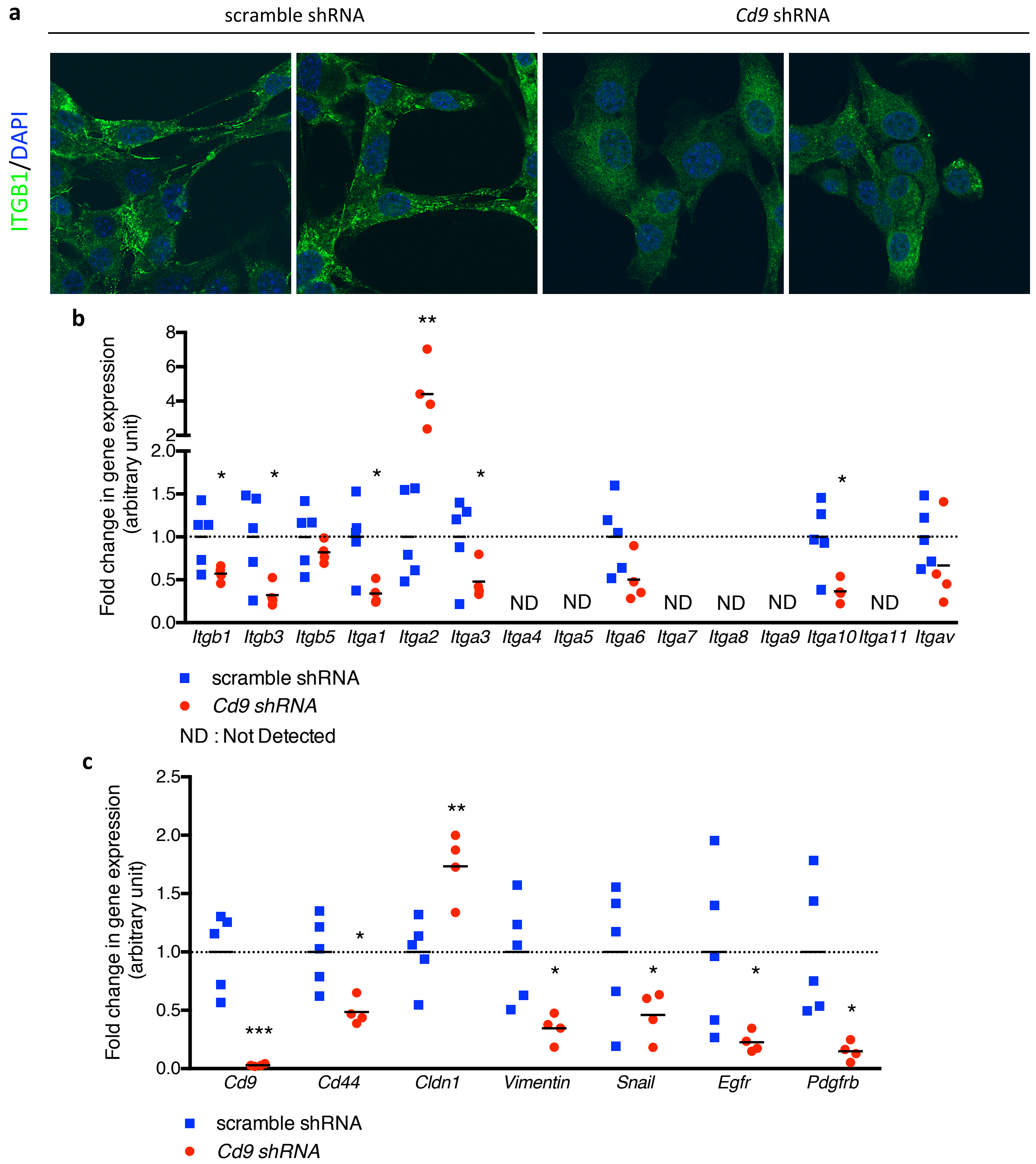

<u>Supplementary figure 13</u>: *In vitro* CD9 deficiency in PEC modifies ITGB1 expression. (a) Representative images of immunofluorescent stainings for ITGB1 (green) in PEC M12 cells transduced with a scramble or a *Cd9* shRNA. Nuclei were counterstained with DAPI (blue). (b,c) Fold change in mRNA expression for (b) *Itgb1, Itgb3, Itgb5, Itga1* to *Itga11* and *Itgav* and (c) *Cd9, Cd44, Cldn1, vimentin, snail, Egfr* and *Pdgfrb* in PEC M12 cells transduced with a scramble shRNA (blue dots) or a Cd9 shRNA (red dots). N= 4 and 5, each dot represent one cell culture well. *T* test: * p<0.05, ** p<0.01 Data represent mean +/- s.e.m. and individual values are shown in dots. Source data are provided as a Source Data file.

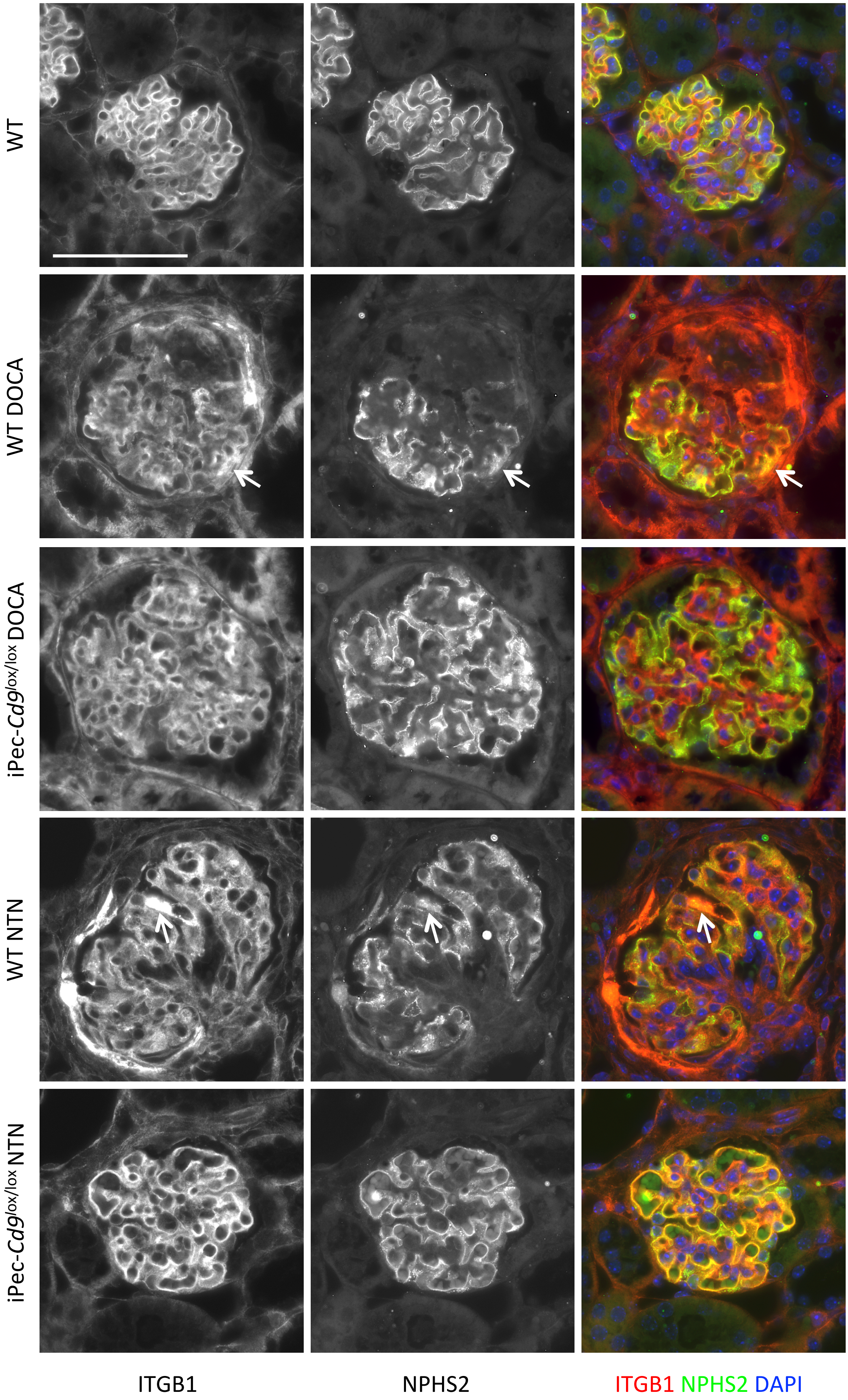
WT
WT DOCA
iPec-Cd9lox/lox DOCA
WT NTN
iPec-Cd9lox/lox NTN
ITGB1
NPHS2
ITGB1 NPHS2 DAPI

<u>Supplementary figure 14</u>: ***De novo* ITGB1 expression is decreased in iPec-*Cd9*$^{lox/lox}$ mice.** Representative images of immunofluorescent stainings for ITGB1 (red) and NPHS2 (green) in iPec-*Cd9*$^{wt/wt}$ (WT), iPec-*Cd9*$^{wt/wt}$ DOCA, iPec-*Cd9*$^{wt/wt}$ NTN, iPec-*Cd9*$^{lox/lox}$ DOCA and iPec-*Cd9*$^{lox/lox}$ NTN mice. Nuclei were counterstained with DAPI (blue). N=6 WT, n=8 DOCA and n=8 NTN mice per genotype. Scale bar: 50 µm.

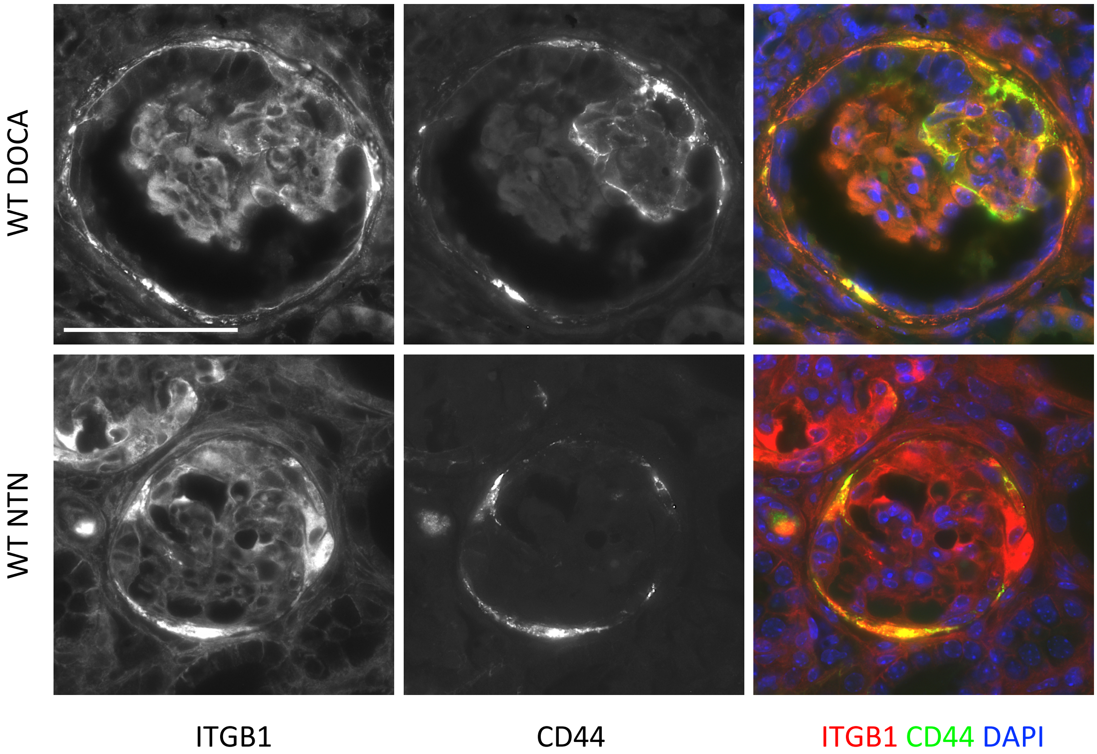

<u>Supplementary figure 15</u>: *De novo* ITGB1 expression in activated PECs in experimental FSGS and CGN. Representative images of immunofluorescent stainings for ITGB1 (red) and CD44 (green) in WT DOCA and WT NTN mice. Nuclei were counterstained with DAPI (blue). N=8 mice per genotype. Scale bar: 50 µm.

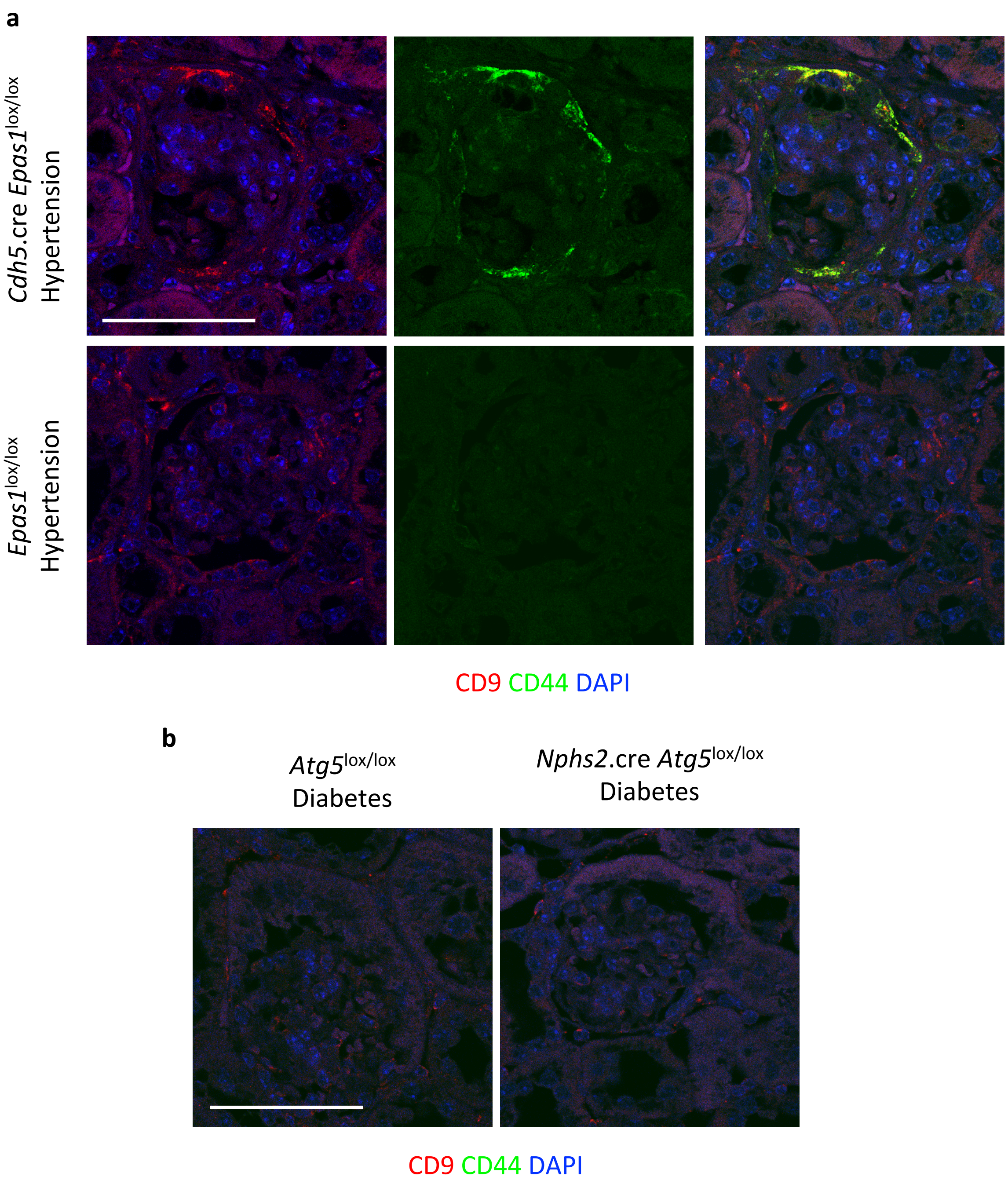

Supplementary figure 16: **CD9 expression in activated CD44+ PECs in experimental FSGS.** (a-b) Representative images of immunofluorescent stainings for CD9 (red) and CD44 (green) in (a) WT (*Epas1*$^{lox/lox}$) and *Cdh5*.cre *Epas1*$^{lox/lox}$ hypertensive mice and (b) WT (*Atg5*$^{lox/lox}$) and *Nphs2*.cre *Atg5*$^{lox/lox}$ diabetic mice. N=6 mice per genotype. Scale bar: 50 µm.

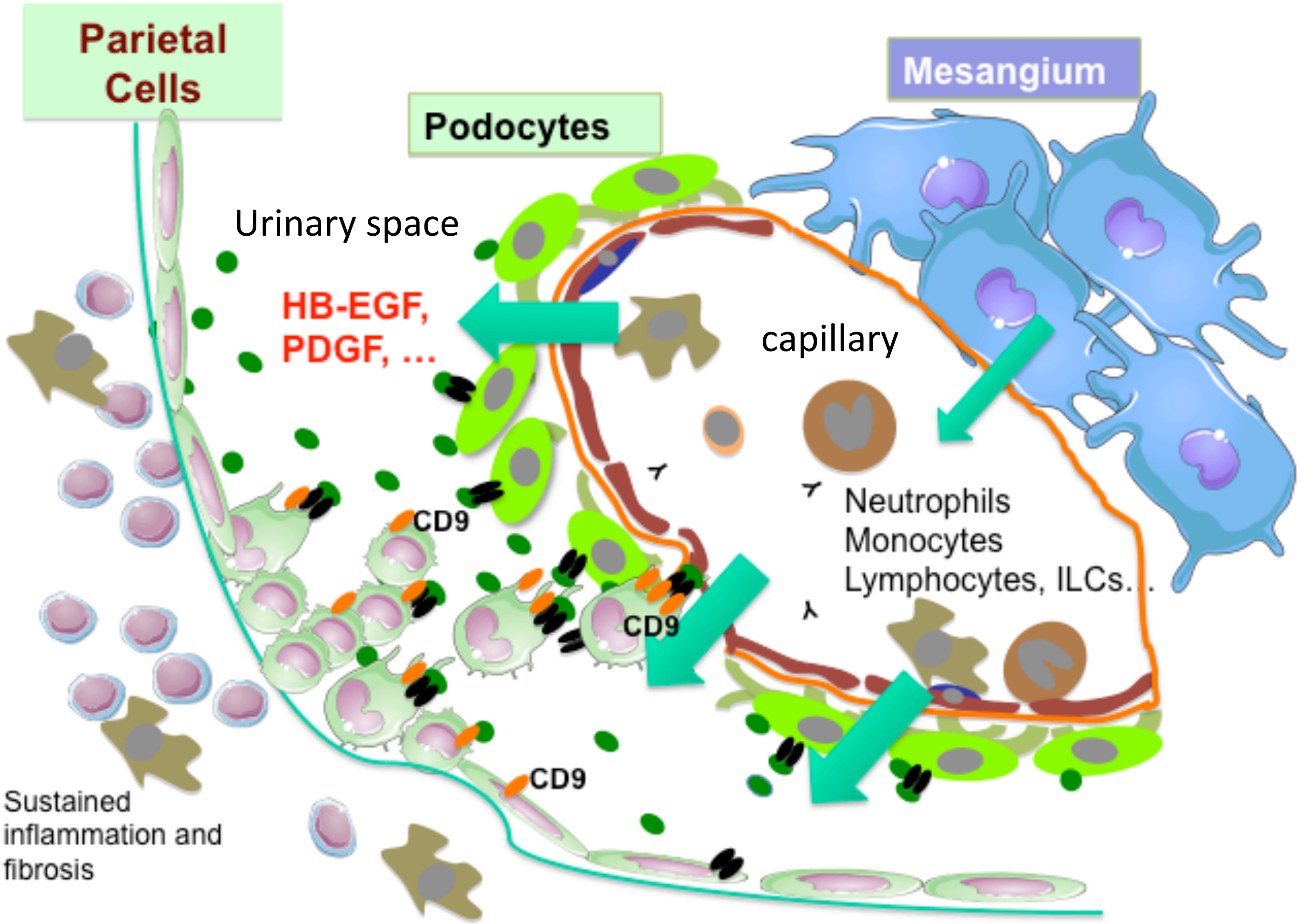

<u>Supplementary figure 17</u> : Mechanistic scheme for the pathogenesis of the destructive recruitment of parietal epithelial cells (PECs) to the glomerular capillary in the context of Focal Segmental Glomerulosclerosis (FSGS) and Crescentic Glomerulonephritis (CGN).

De novo expression of CD9 (orange ovals) in PECs is hallmark of human and mouse extracapillary diseases characterized by pathogenic PEC (pale green) recruitment to the glomerular tuft. This study suggests that CD9-dependent molecular complex adjusts the threshold for proliferation and directional migration of these cells toward gradient (green arrows) of capillary-derived chemoattractants (such as PDGFBB and HB-EGF). This mechanism unravels the crucial role of PECs for development of such severe glomerular diseases.

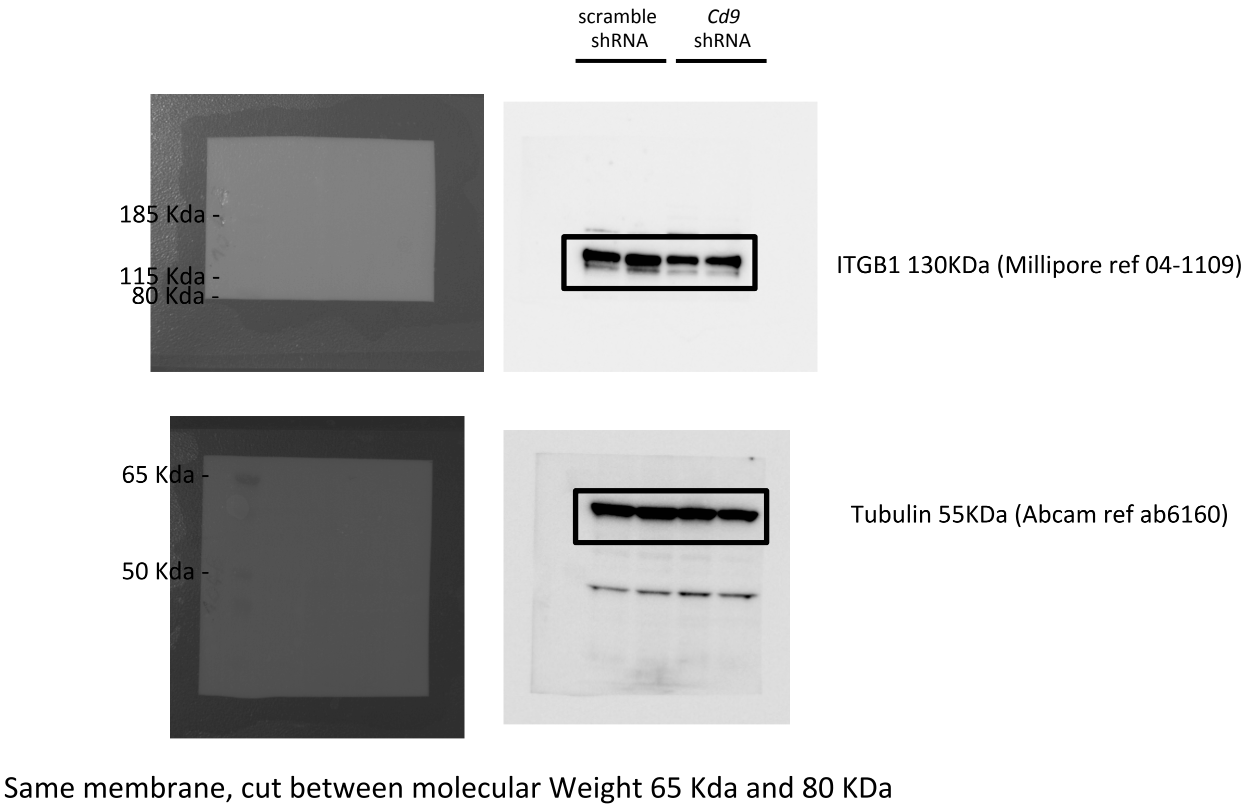

Supplementary Figure 18: Uncropped gel for figure 6A. Western blot analysis of the expression of ITGB1 and Tubulin in PEC M12 cells transduced with a control shRNA or a *Cd9* shRNA. Anti-ITGB1 antibody from Millipore (ref 04-1109) and anti-tubulin antibody from Abcam (ref ab6160) were used for blotting.

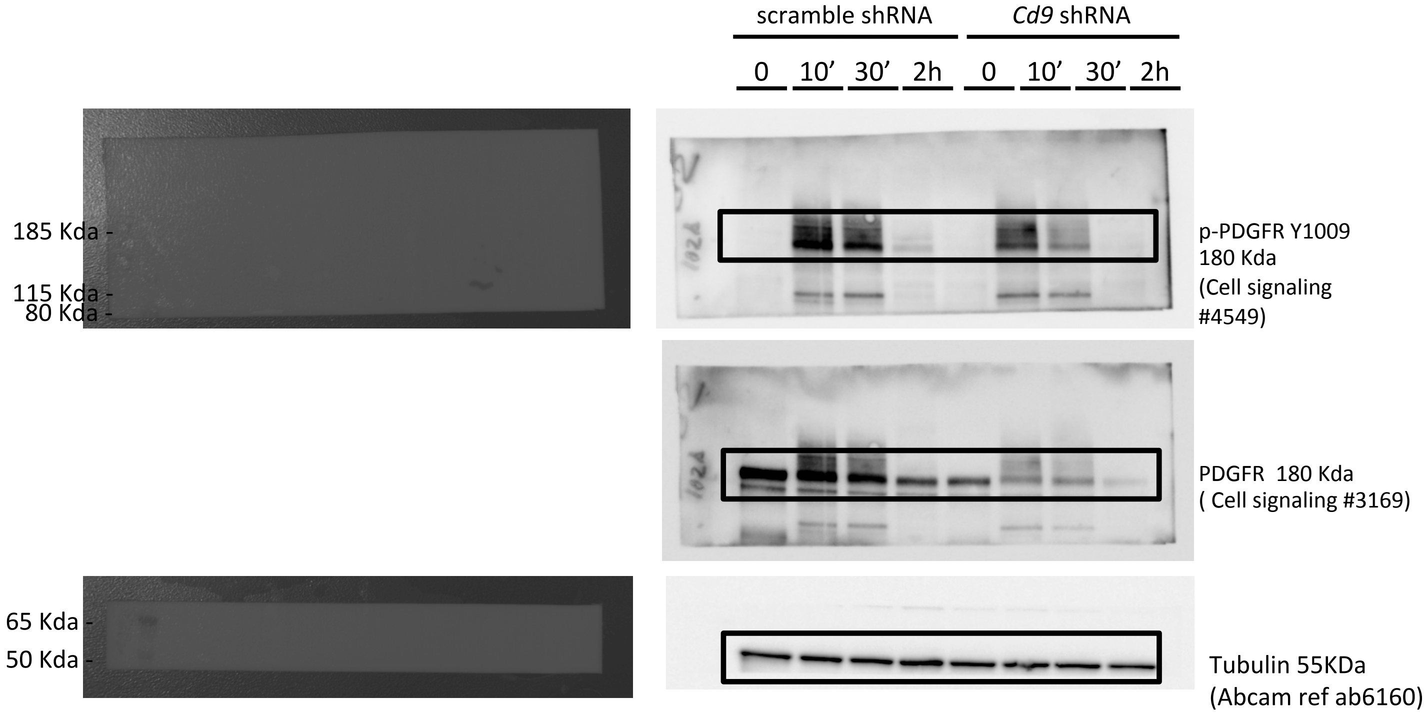

Same membrane, cut between molecular Weight 65 Kda and 80 KDa

<u>Supplementary Figure 19: Uncropped gel for figure 6D upper panel.</u> Western blot analysis of the expression of phospho PDGFR (Y1009), total PDGFR and Tubulin in PEC M12 cells transduced with a control shRNA or a *Cd9* shRNA along the time course of PDGF stimulation. Anti-phospho PDGFR (Y1009) antibody from Cell signaling technology (ref #4549), anti-PDGFR from Cell signaling technology (ref #3169) and anti-tubulin antibody from Abcam (ref ab6160) were used for blotting.

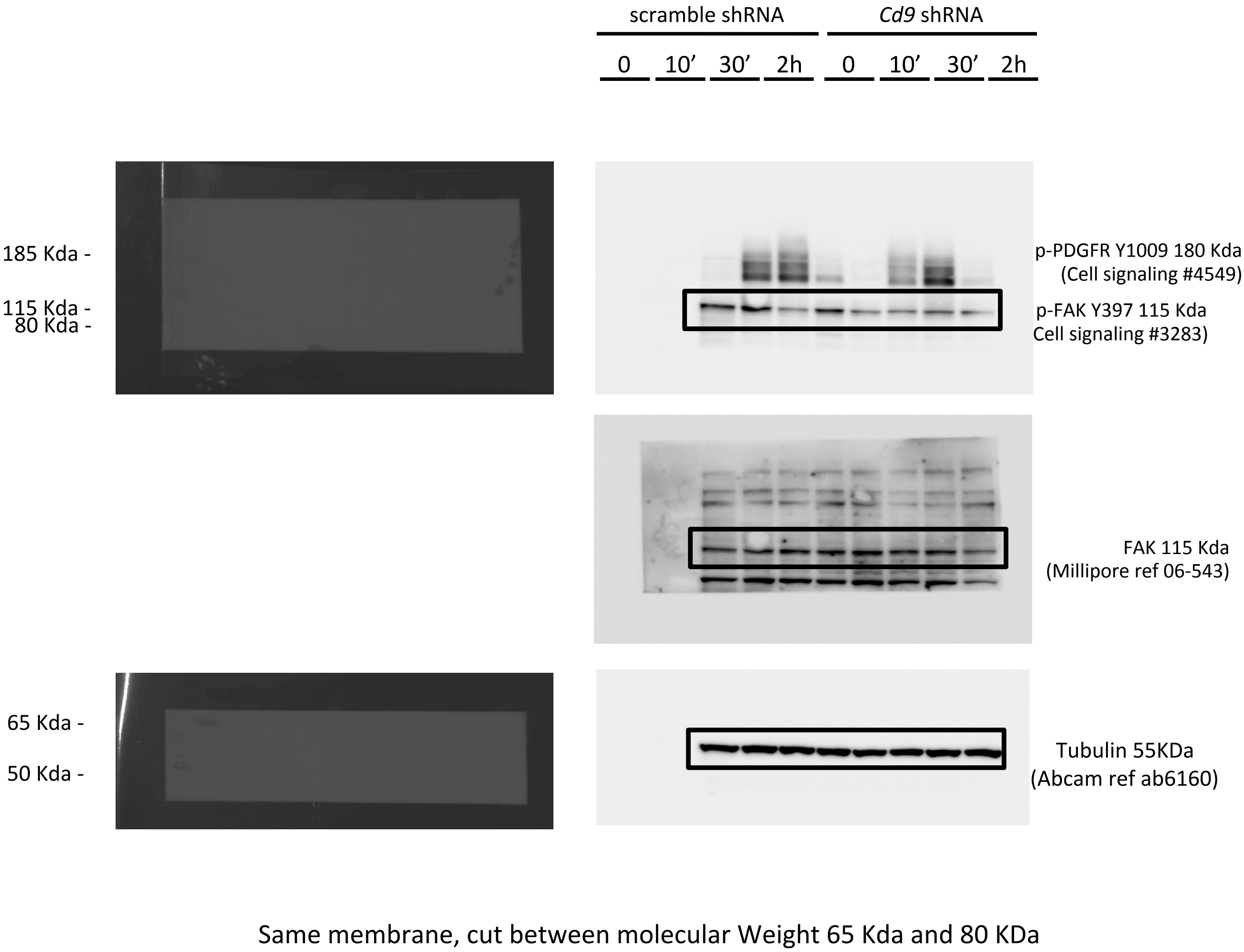

Supplementary Figure 20: Uncropped gel for figure 6D lower panel. Western blot analysis of the expression of phospho FAK (Y397), total FAK and Tubulin in PEC M12 cells transduced with a control shRNA or a *Cd9* shRNA along the time course of PDGF stimulation. Anti-phospho FAK (Y397) antibody from Cell signaling technology (ref #3283), anti-FAK from Millipore (ref 06-543) and anti-tubulin antibody from Abcam (ref ab6160) were used for blotting.

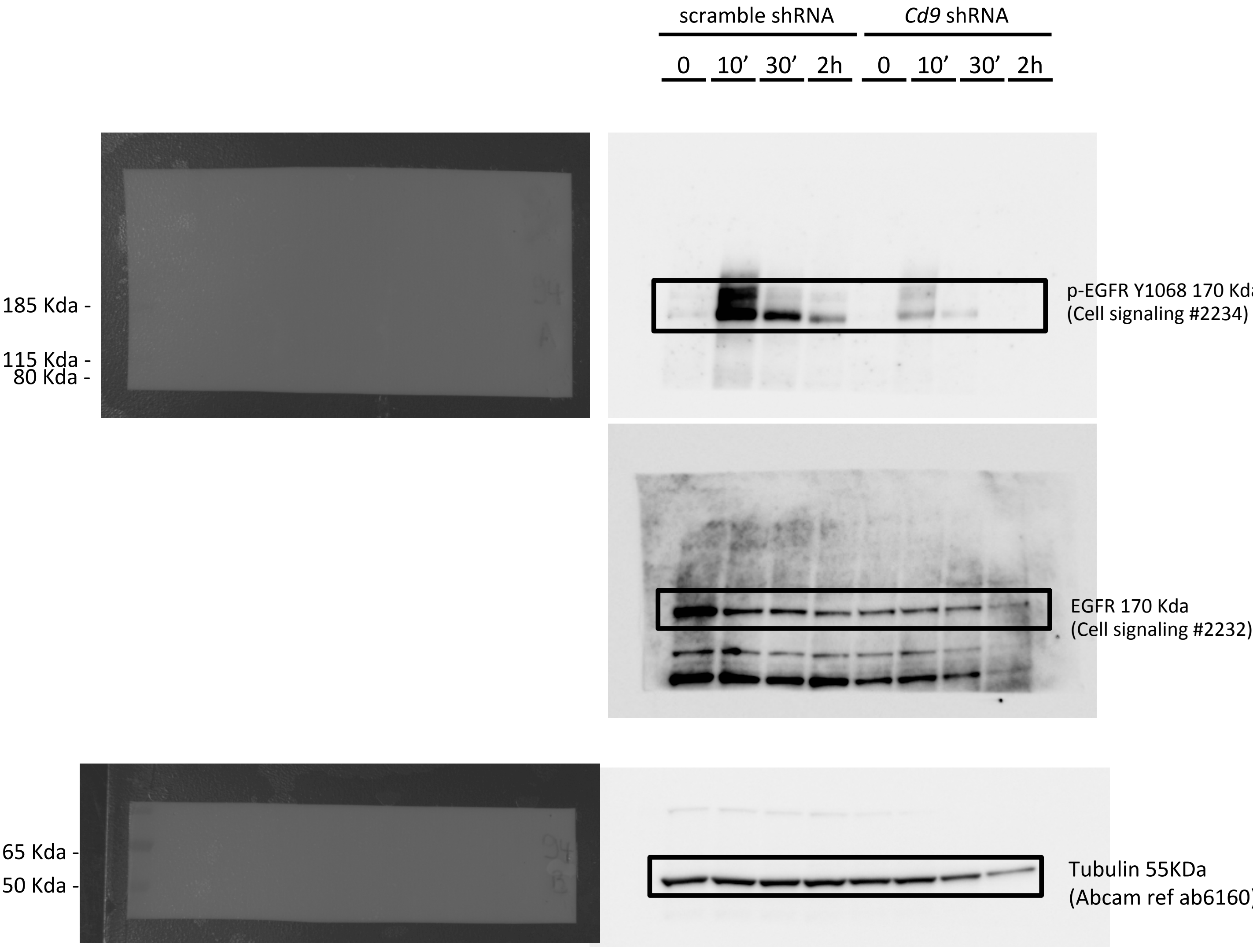

Same membrane, cut between molecular Weight 65 Kda and 80 KDa

Supplementary Figure 21: Uncropped gel for figure 6H upper panel. Western blot analysis of the expression of phospho EGFR (Y1068), total EGFR and Tubulin in PEC M12 cells transduced with a control shRNA or a *Cd9* shRNA along the time course of HB-EGF stimulation. Anti-phospho EGFR (Y1068) antibody from Cell signaling technology (ref #2234), Anti-EGFR antibody from Cell signaling technology (ref #2232) and anti-tubulin antibody from Abcam (ref ab6160) were used for blotting.

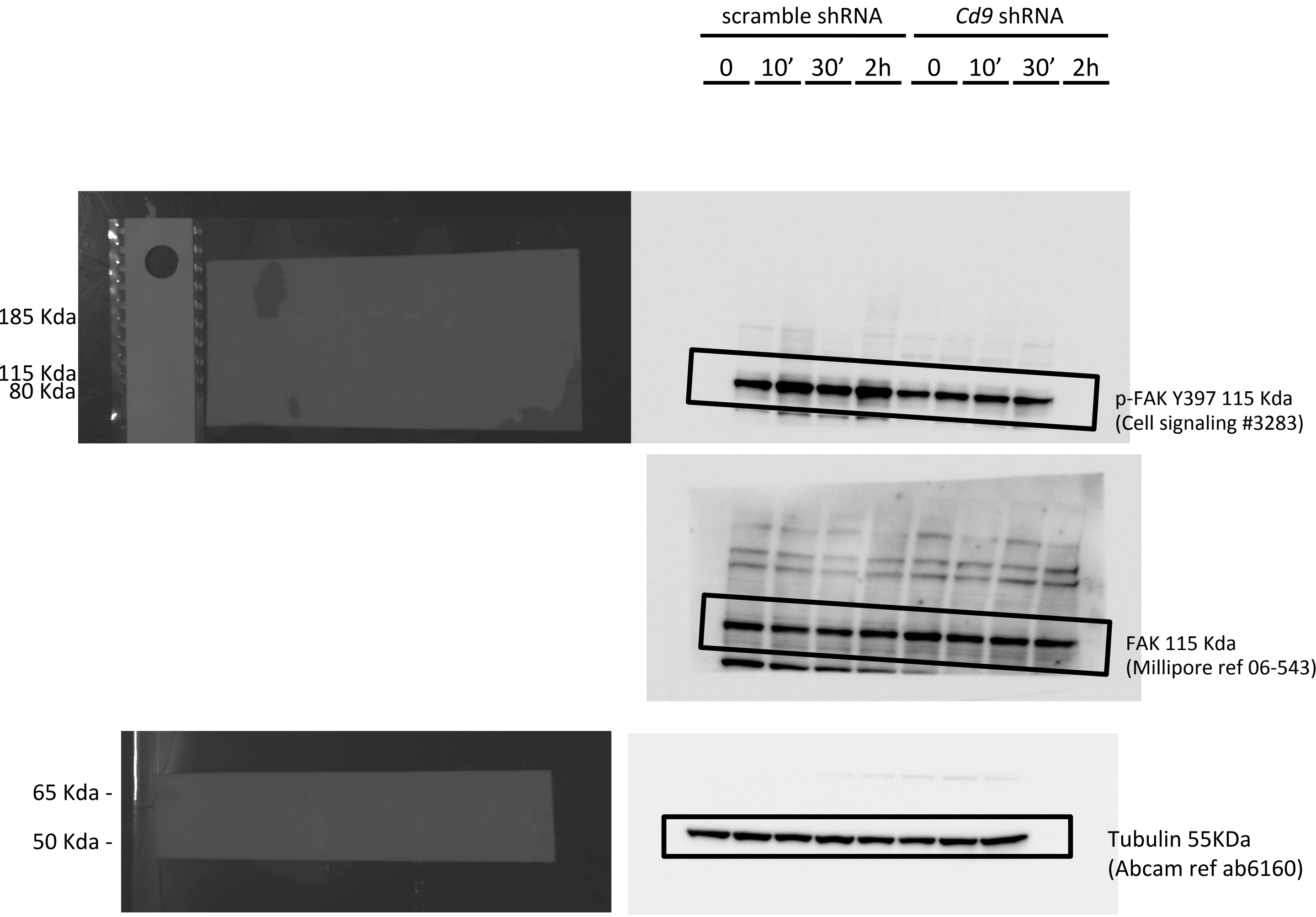

Same membrane, cut between molecular Weight 65 Kda and 80 KDa

Supplementary Figure 22: Uncropped gel for figure 6H lower panel. Western blot analysis of the expression of phospho FAK (Y397), total FAK and Tubulin in PEC M12 cells transduced with a control shRNA or a *Cd9* shRNA along the time course of PDGF stimulation. Anti-phospho FAK (Y397) antibody from Cell signaling technology (ref #3283), anti-FAK from Millipore (ref 06-543) and anti-tubulin antibody from Abcam (ref ab6160) were used for blotting.

Supplementary table 1 : Human kidney biopsies : diagnosis, patient characteristics and CD9-ITGB1 staining

Gender	Age	Diagnosis	Relapse	Glomerular CD9 expression	Colocalization CD9 / ITGB1
Female	45	Normal kidney		-	-
Male	28	Normal kidney transplant		-	-
Male	61	Minimal change disease	First episode	-	-
Male	62	Minimal change disease	First episode	-	-
Female	44	Focal Segmental Glomerulosclerosis	First episode	+	+
Female	51	FSGS- Sickle Cell disease	First episode	+	+
Male	35	IgA nephropathy with FSGS	First episode	+	+
Male	31	Focal Segmental Glomerulosclerosis	First episode	+	+
Female	29	Collapsing FSGS – fibrous kidney	Relapse	-	-
Male	43	Granulomatosis with polyangiitis	First episode	+	+
Female	44	Granulomatosis with polyangiitis	First episode	+	+
Male	48	Granulomatosis with polyangiitis	First episode	+	+
Female	83	Micro-polyangiitis ANCA negative	Relapse	+	+
Male	54	Micro-polyangiitis MPO-ANCA-positive	First episode	+	+
Male	80	Micro-polyangiitis MPO-ANCA-positive	First episode	+	+
Female	73	Micro-polyangiitis MPO-ANCA-positive	Relapse	+	+
Female	75	Micro-polyangiitis MPO-ANCA-positive	Relapse	+	+
Female	65	Micro-polyangiitis PR3-ANCA-positive	First episode	+	+

Supplementary Table 2: Additional human kidney biopsies: diagnosis, patient characteristics and CD9-staining

Gender	Age	Diagnosis	Relapse	Glomerular CD9 expression	CD9 cellular expression in glomeruli
**F**	24	FSGS	First episode	+	PEC – podocytes
**F**	24	FSGS	Relapse	+	PEC and floculocapsular synechia
**F**	28	FSGS	First episode	+	All PEC – podocytes
**F**	39	FSGS	First episode	+	
**M**	35	FSGS	First episode	+	Podocytes
**F**	54	HIVAN	First episode	+	
**M**	39	IgA nephropathy	First episode	+	Crescent
**M**	48	IgA nephropathy	First episode	+	Crescent – podocytes – PEC
**F**	20	IgA nephropathy	First episode	+	Podocytes – PEC – floculocapsular synechia
**M**	43	IgA nephropathy	First episode	+	PEC – floculocapsular synechia
**M**	55	IgA nephropathy	First episode	-	-
**M**	24	IgA nephropathy	First episode	-	-
**F**	33	IgA nephropathy	First episode	+	
**M**	30	IgA nephropathy	First episode	+	Sclerotic glomerulus
**M**	40	IgA nephropathy with secondary FSGS	First episode	+	Podocytes – PEC – floculocapsular synechia
**M**	33	IgA nephropathy with secondary FSGS	First episode	+	Normal PEC
**M**	27	IgA nephropathy with secondary FSGS	First episode	+	Podocytes – PEC – floculocapsular synechia
**M**	61	IgA nephropathy with secondary FSGS	First episode	+	Floculocapsular synechia
**F**	35	IgA nephropathy with secondary FSGS	First episode	+	Sclerotic glomeruli
**M**	27	IgA nephropathy with secondary FSGS	First episode	+	Podocytes
**F**	26	Class IV Lupus nephritis	First episode	+	Podocytes – PEC
**M**	69	ANCA-negative vasculitis with secondary FSGS	Relapse	+	Crescent – floculocapsular synechia
**F**	41	ANCA-negative vasculitis	First episode	+	Crescent – podocytes – PEC
**F**	59	ANCA-positive vasculitis	Relapse	+	Crescent – podocytes – PEC
**F**	67	MPO-ANCA-positive vasculitis	First episode	+	Crescent – podocytes – PEC
**M**	94	MPO-ANCA-positive vasculitis	First episode		Crescent – podocytes
**F**	53	MPO-ANCA-positive vasculitis	First episode	+	Crescent – PEC – podocytes
**M**	30	PR3-ANCA-positive vasculitis	First episode	+	Crescent – podocytes – PEC and floculocapsular synechia
**M**	49	PR3-ANCA-positive vasculitis	First episode	+	Crescent – PEC - podocytes
**F**	24	PR3-ANCA-positive vasculitis with secondary FSGS	Relapse	+	PEC – podocytes and floculocapsular synechia
**M**	46	PR3-ANCA-positive vasculitis	First episode	+	Crescent – PEC – podocytes and floculocapsular synechia

Supplementary Table 3: qPCR primers

Mm-Ppia F	CACCGTGTTCTTCGACATCA
Mm-Ppia R	CAGTGCTCAGAGCTCGAAAGT
Mm-Vimentin F	CCAACCTTTTCTTCCCTGAA
Mm-Vimentin R	TGAGTGGGTGTCAACCAGAG
Mm-Snai1 F	CTTGTGTCTGCACGACCTGT
Mm-Snai1 R	AGTGGGAGCAGGAGAATGG
Mm-Cd44 F	AGCTGACGAGACCCGGAATC
Mm-Cd44 R	TCCCAGGAATGACGTCTCCA
Mm-Cldn1 F	GATGTGGATGGCTGTCATTGG
Mm-Cldn1 R	CCATGCTGTGGCCACTAATGT
Mm-Pdgfrb F	AACAGAAGACAGCGAGGTGG
Mm-Pdgfrb R	TGGTATCACTCCTGGAAGCC
Mm-Egfr F	ATCCTCTGCAGGCTCAGAAA
Mm-Egfr R	GGCGTTGGAGGAAAAGAAAG
Mm-Cd9 F	TGCAGTGCTTGCTATTGGAC
Mm-Cd9 R	GGCGAATATCACCAAGAGGA
Mm-Itga1 F	ATGACGCTCTGCCAAACTCA
Mm-Itga1 R	TGTTGTACGCACTGTCTCCC
Mm-Itga2 F	ACCCACGGAGAAAGCAGAAG
Mm-Itga2 R	CGCCGATGGTTTAGCTGTTG
Mm-Itga3 F	CCTAGCCGAGGTCCAAAGAG
Mm-Itga3 R	TCCGTTTTCCAGATCCGTGG
Mm-Itga4 F	TGAAGCATCCCTGGCCACTA
Mm-Itga4 R	GGTCCAGGTTGTAGGAGTGC
Mm-Itga5 F	CATTCGCCTCTGGGAGGTTT
Mm-Itga5 R	CACACTGACTCCGTCCCTTC
Mm-Itga6 F	AGTTTGGCTCCTGTCAGCAA
Mm-Itga6 R	ACGGACGATCCCTTTCCAGT
Mm-Itga7 F	CTACCTCGTCTTTGGGGCTC
Mm-Itga7 R	ATTCAAGGTCAAGTCTCCGGC
Mm-Itga8 F	CGCACAGGGCGAGAGG
Mm-Itga8 R	CAAGTTGAACGCCAGACACG
Mm-Itga9 F	GGGCTTAGACTCGGAGCGT
Mm-Itga9 R	ATCTCCGGTCAGGGTTGGTA
Mm-Itga10 F	GGCTGCTGGTAGTTGTCACT
Mm-Itga10 R	ACCGCAATCCCATAACGTGT
Mm-Itga11 F	CCCCATCTGGATCATCGTGG
Mm-Itga11 R	CTCTTGCGCTTGGCACTTTT
Mm-Itgav F	CGTCCTCCAGGATGTTTCTCC
Mm-Itgav R	CCAAACCACTGGTGGGACTT
Mm-Itgb1 F	GGACGCTGCGAAAAGATGAA
Mm-Itgb1 R	CCACAATTTGGCCCTGCTTG
Mm-Itgb3 F	AGTGGCCGGGACAACTCT
Mm-Itgb3 R	GGACTCTCCAACAACAACGC
Mm-Itgb5 F	GCCCGTTATGAAATGGCCTC
Mm-Itgb5 R	AGGCGAAATCGACAGTGTGT
