# Supplementary material for: The tetraspanin CD9 controls migration and proliferation of parietal epithelial cells and glomerular disease progression
Source: Nat Commun. 2019 Jul 24;10:3303. doi: 10.1038/s41467-019-11013-2 (PMC6656772; doi:10.1038/s41467-019-11013-2)
Supplement: Supplementary file 3 — Description of Additional Supplementary Files [file 41467_2019_11013_MOESM3_ESM.docx]

**Description of Supplementary Files**

**File Name:** **Supplementary Movie 1.**

**Description:** Movie of PEC migration in ibidi chambers for migration of Cd9 shRNA PEC in basal conditions (BL). 24h migration in 5 sec movie (1h is 208 msec).

**File Name: Supplementary Movie 2.**

**Description:** Movie of PEC migration in ibidi chambers for migration of Cd9 shRNA PEC after stimulation by HB-EGF during 24 hours. 24h migration in 5 sec movie (1h is 208 msec).

**File Name:** **Supplementary Movie 3.**

**Description:** Movie of PEC migration in ibidi chambers for migration of Cd9 shRNA PEC after stimulation by PDGF-BB during 24 hours. 24h migration in 5 sec movie (1h is 208 msec).

**File Name:** **Supplementary Movie 4.**

**Description:** Movie of PEC migration in ibidi chambers for migration of scramble shRNA PEC in basal conditions (BL). 24h migration in 5 sec movie (1h is 208 msec).

**File Name:** **Supplementary Movie 5.**

**Description:** Movie of PEC migration in ibidi chambers for migration of scramble shRNA PEC after stimulation by HB-EGF during 24 hours. 24h migration in 5 sec movie (1h is 208 msec).

**File Name:** **Supplementary Movie 6.**

**Description:** Movie of PEC migration in ibidi chambers for migration of scramble shRNA PEC after stimulation by PDGF-BB during 24 hours. 24h migration in 5 sec movie (1h is 208 msec).
