# Supplementary material for: The tetraspanin CD9 controls migration and proliferation of parietal epithelial cells and glomerular disease progression
Source: Nat Commun. 2019 Jul 24;10:3303. doi: 10.1038/s41467-019-11013-2 (PMC6656772; doi:10.1038/s41467-019-11013-2)
Supplement: Supplementary file 10 — Reporting Summary [file 41467_2019_11013_MOESM10_ESM.pdf]

nature research

Corresponding author(s): Carole Henique
Pierre-Louis Tharaux

Last updated by author(s): Jun 7, 2019

Reporting Summary

Nature Research wishes to improve the reproducibility of the work that we publish. This form provides structure for consistency and transparency in reporting. For further information on Nature Research policies, see Authors & Referees and the Editorial Policy Checklist.

Statistics

For all statistical analyses, confirm that the following items are present in the figure legend, table legend, main text, or Methods section.

n/a	Confirmed	
☐	☒	The exact sample size (n) for each experimental group/condition, given as a discrete number and unit of measurement
☐	☒	A statement on whether measurements were taken from distinct samples or whether the same sample was measured repeatedly
☐	☒	The statistical test(s) used AND whether they are one- or two-sided<br>*Only common tests should be described solely by name; describe more complex techniques in the Methods section.*
☐	☒	A description of all covariates tested
☐	☒	A description of any assumptions or corrections, such as tests of normality and adjustment for multiple comparisons
☐	☒	A full description of the statistical parameters including central tendency (e.g. means) or other basic estimates (e.g. regression coefficient) AND variation (e.g. standard deviation) or associated estimates of uncertainty (e.g. confidence intervals)
☒	☐	For null hypothesis testing, the test statistic (e.g. F, t, r) with confidence intervals, effect sizes, degrees of freedom and P value noted<br>*Give P values as exact values whenever suitable.*
☒	☐	For Bayesian analysis, information on the choice of priors and Markov chain Monte Carlo settings
☒	☐	For hierarchical and complex designs, identification of the appropriate level for tests and full reporting of outcomes
☐	☒	Estimates of effect sizes (e.g. Cohen's d, Pearson's r), indicating how they were calculated

Our web collection on statistics for biologists contains articles on many of the points above.

Software and code

Policy information about availability of computer code

Data collection	Tail cuff plethysmography: BP-2000 Blood pressure analyzing system from Visitech system<br>Western blot acquisition: LAS-4000 software, Burlington<br>Flow cytometry acquisition: LSRII, BD bioscience<br>Ibidi chamber picture acquisition: NIS-Elements AR software
Data analysis	Statistics: Prism 6.0, GraphPad software<br>Flow cytometry: FlowJo software, LLC<br>Migration assay in ibidi chamber quantiication: NIS-Elements AR software, v4.60.00<br>Fiji Image processing software (NIH)

For manuscripts utilizing custom algorithms or software that are central to the research but not yet described in published literature, software must be made available to editors/reviewers. We strongly encourage code deposition in a community repository (e.g. GitHub). See the Nature Research guidelines for submitting code & software for further information.

Data

Policy information about availability of data

All manuscripts must include a data availability statement. This statement should provide the following information, where applicable:

- Accession codes, unique identifiers, or web links for publicly available datasets
- A list of figures that have associated raw data
- A description of any restrictions on data availability

The authors declare that the data supporting the findings of this study are available within the paper and its supplementary information files. Source data for figures 1b, 2a-b, 3a-b,d, 4c-d,g-i, 5a-b,d,h,i, and 6b,e-k and supplementary figures are provided as a Source Data file. All data are available from the corresponding authors upon reasonable request.

Field-specific reporting

Please select the one below that is the best fit for your research. If you are not sure, read the appropriate sections before making your selection.

☒ Life sciences ☐ Behavioural & social sciences ☐ Ecological, evolutionary & environmental sciences

For a reference copy of the document with all sections, see nature.com/documents/nr-reporting-summary-flat.pdf

Life sciences study design

All studies must disclose on these points even when the disclosure is negative.

Sample size	Sample size was chosen in accordance with similar published experiments by other independent groups as well as our lab
Data exclusions	No data exclusion
Replication	Animal experiments were reproduced 2 to 3 and reproductibility of results was confirmed.
Randomization	Allocation was random between placebo and treated mice in all experiments.
Blinding	Histological quantifications, biochemical measurement, Western blot quantification, cell migration assay and flow cytometry analysis were done blindly

Reporting for specific materials, systems and methods

We require information from authors about some types of materials, experimental systems and methods used in many studies. Here, indicate whether each material, system or method listed is relevant to your study. If you are not sure if a list item applies to your research, read the appropriate section before selecting a response.

Materials & experimental systems

n/a	Involved in the study	
☐	☒	Antibodies
☐	☒	Eukaryotic cell lines
☒	☐	Palaeontology
☐	☒	Animals and other organisms
☐	☒	Human research participants
☒	☐	Clinical data

Methods

n/a	Involved in the study	
☒	☐	ChIP-seq
☐	☒	Flow cytometry
☒	☐	MRI-based neuroimaging

Antibodies

Antibodies used	Flow cytometry: phycoerythrin (PE)-conjugated anti-human Ki67 mAb (clone B56) (BD Pharmingen)<br>Western blot: rat anti-CD9 (BD Pharminogen, 553758, 1 :1000), rabbit anti-phospho-PDGF receptor-ß Y1009 (Cell Signaling Technology, 4549 ; 1:1000), rabbit anti-PDGFRß (Cell Signaling, 3169; 1:1000), rabbit anti-integrin ß1 (Millipore, 04-1109, 1:1000), rabbit anti-CASP3 (Cell Signaling Technology, 9662; 1:1000), rabbit anti-phospho EGFR Y1068 (Cell Signaling Technology, 2234; 1:1000), rabbit anti EGFR (Cell Signaling Technology, 2232 ; 1:1000), rabbit anti-phospho-FAK Y397 (Cell Signaling 3283, 1 :1000), rabbit anti-FAK (Millipore, 06-543,1:1000). Secondary antibodies: horseradish peroxidase-conjugated secondary antibodies (Cell Signaling Technology, 7074 and 7076; 1:2000; Jackson Immunoresearch, 706-036-148; 1:1000)<br>Immunohistochemistry: rabbit anti-WT1 (Abcam, ab89901, 1:100), goat anti-PODXL (R&D systems, BAF1556, 1:200), rabbit anti-CD9 (Abcam, ab92726, 1:100 and C Boucheix home made), rat anti-CD44 (Abcam, ab119348, 1:100), rat anti-CD44 AF647-conjugated (Biolegend, 103018, 1:100), rabbit anti-integrin ß1 (Abcam, ab179471, 1:500), goat anti–synaptopodin antibody (Santa Cruz Biotechnology; SC21537; 1:400), rabbit anti–p57 antibody (Santa Cruz Biotechnology; SC8298; Santa Cruz; 1:200). Secondary antibodies: donkey anti-goat IgG (H+L) AF488-conjugated antibody (Life Technologies, A-11005, 1:400), donkey anti-rabbit IgG (H+L) AF594-conjugated antibody (A-21207, Life Technologies, 1:400), donkey anti-rat IgG (H+L) AF488-conjugated antibody (A-21208, Life Technologies, 1:400), donkey anti-rabbit IgG(H+L) Cy5-conjugated antibody (Jackson Immunoresearch, 1:200).
Validation	All primary antibodies used for Western blot were choosen because validated by the manufacturer and already published elsewhere to be validated for such application in mouse. All these antibodies gave a specific band at the good molecular weight. All primary antibodies used for IHC, except the human anti-CD9 home-made antibody, were validated by the manufacturer, extensively used in the litterature and gave specific signal (i.e, podocyte nuclei for WT1 and P57, podocyte membranes for PODXL and synaptododin, membranes for ITGB1, PEC and tubule membranes for CD44).

Eukaryotic cell lines

Policy information about cell lines

Cell line source(s)	PEC M12 cells: primary parietal epithaliel cell line from M Moeller
Authentication	PEC M12 cells were authentified previously by Marcus Moeller lab. Western blot for PEC markers were performed in this study to validate authenticity of the cell line.
Mycoplasma contamination	Not tested for mycoplasma in the present study
Commonly misidentified lines (See ICLAC register)	*Name any commonly misidentified cell lines used in the study and provide a rationale for their use.*

Animals and other organisms

Policy information about studies involving animals; ARRIVE guidelines recommended for reporting animal research

Laboratory animals	mus musculus, C57BL6/J 129S2 mixed genetic background. Male from 10 to 15 weeks-old at the begining of the experiment. Cd9 KO, Nphs2.Cre Cd9flox/flox, Pf4.Cre Cd9flox/flox, pPEC-rtTA-M2.cre Cd9flox/flox
Wild animals	the study did not involve wild animals
Field-collected samples	the study did not involve samples collected from the field
Ethics oversight	All animal procedures were performed in accordance with guidelines of the European Community (L358–86/609EEC), and were approved by the Institut National de la Santé et de la Recherche Médicale and the Ministry for Higher Education and Research (MESR 7646 and TARGET GLOMDIS).

Note that full information on the approval of the study protocol must also be provided in the manuscript.

Human research participants

Policy information about studies involving human research participants

Population characteristics	Gender, age and diagnosis of each patient are reported in supplementary table 1 and 2. 49 biospsies from 49 patients (age 24-83 years-old, 23 females)
Recruitment	Human tissue was used after informed consent by the patients and approval form, and following the guidelines of the local Ethics Committee (IRB00003888, FWA00005831).
Ethics oversight	Human tissue was used after informed consent by all the patients and approval form, and kidney biopsies collection was approved by the Inserm Ethics Committee (IRB00003888, FWA00005831 NIH OHRP, Office of Human Research Protection). Kidney biopsy specimens were collected in compliance with all relevant ethical regulations and those with sufficient tissue for immunohistochemical evaluation after the completion of diagnosis workup were included.

Note that full information on the approval of the study protocol must also be provided in the manuscript.

Flow Cytometry

Plots

Confirm that:

☒ The axis labels state the marker and fluorochrome used (e.g. CD4-FITC).

☒ The axis scales are clearly visible. Include numbers along axes only for bottom left plot of group (a 'group' is an analysis of identical markers).

☒ All plots are contour plots with outliers or pseudocolor plots.

☒ A numerical value for number of cells or percentage (with statistics) is provided.

Methodology

Sample preparation	Cells viability was assessed using APC conjugated Annexin-V (ImmunoTools) combined with propidium iodide (PI)(Thermo Fisher Scientific). Cells were plated in 6-well plates during 24 hours. Cells were then trypsinized and washed in cold phosphate-buffered saline (PBS w/o Ca2+). Supernatants were kept and included in analysis. After centrifugation, cells were then resuspended in 100 ul of Annexin-V binding buffer (BD Pharmingen) and directly stained with Annexin-V and PI at room temperature for 5 min in the dark. A control tube without Annexin-V binding buffer was used to sustain aspecific signal from final analysis. Samples were analysed on a BD LSRII flow cytometer (BD Bioscience). Data were analyzed using FlowJo software (FlowJo, LLC). Viable cells were defined as both Annexin-V and PI negative, apoptotic cells as Annexin-V positive and PI negative, necrotic cells as both Annexin-V and PI positive and all populations were expressed as percentage of total cells. To assess cell proliferation, we used the nuclear antigen Ki67 staining. After overnight starvation in ECBM+1%FBS, cells were

treated 24 hours with PDGF-BB (10 ng/mL) or HBEGF (20 ng/mL) or corresponding controls. Cells were then trypsinized and washed in cold PBS. Supernatants were kept and included in analysis. After centrifugation, cells were permeabilized with 1 mL of ice cold ethanol for 2h at -20˚C. Following two washes with FACS buffer (HBSS, 2% FBS and 10mM HEPES), cells were stained for 30 min at room temperature in the dark with phycoerythrin (PE)-conjugated anti-human Ki67 mAb (clone B56) (BD Pharmingen) and washed. Samples were analyzed on a BD LSRII flow cytometer (BD Bioscience). An isotype control staining with PE mouse IgG1 was performed. Data were analyzed using FlowJo software (FlowJo, LLC).

Instrument	BD LSRII flow cytometer (BD Bioscience)
Software	FlowJo software (FlowJo, LLC)
Cell population abundance	no post-sort fractions
Gating strategy	Viable cells were defined as both Annexin-V and PI negative, apoptotic cells as Annexin-V positive and PI negative, necrotic cells as both Annexin-V and PI positive and all populations were expressed as percentage of total cells.

☒ Tick this box to confirm that a figure exemplifying the gating strategy is provided in the Supplementary Information.
